# Morphometric variability of Carabidae as an indicator of ecological restoration of revitalized habitats in the European important protected landscape area of the Danube floodplains

**DOI:** 10.7717/peerj.21556

**Published:** 2026-07-29

**Authors:** Vladimír Langraf, Kornélia Petrovičová, Viktor Brygadyrenko

**Affiliations:** 1 Department of Zoology and Anthropology, Faculty of Natural Sciences and Informatics, Constantine the Philosopher University in Nitra, Nitra, Slovakia; 2 Faculty of Agrobiology and Food Resources, Slovak University of Agriculture in Nitra, Nitra, Slovakia; 3 Department of Parasitology and Veterinary Expertise, Dnipro State Agrarian and Economic University, Dnipro, Dnipropetrovsk Region, Ukraine; 4 Department of Biodiversity and Ecology, Oles Honchar Dnipro National University, Dnipro, Ukraine, Ukraine

**Keywords:** Carabidae, Ground beetle assemblages, Ellipsoid biovolume, Morphometric variability, Neural networks, Floodplain forest, Poplar nursery

## Abstract

Revitalization measures currently represent an important tool in ecological practice for the recovery of disturbed habitats, and their effectiveness is increasingly assessed using bioindicator groups, including ground beetles (Carabidae). Between 2020 and 2023, we conducted research in the European habitat of the Danube Floodplains, where we evaluated the impact of revitalization measures on ground beetle assemblages. The study was carried out at 15 study sites representing eight habitat types. At each site, five pitfall traps were installed in a linear arrangement. In total, 5,292 individuals belonging to 47 species were analyzed, with a mean ellipsoid biovolume of 192 mm^3^ per individual. PCA analysis revealed a division of species into two main clusters according to their preference for forest and open habitats, with reference sites without management characterized by a higher proportion of apterous and brachypterous species. In contrast, habitats subjected to restoration measures showed dominance of macropterous species typical of disturbed and dynamic ecosystems. Significant differences in ellipsoid biovolume and all morphometric traits were confirmed among habitats and study years. Habitats with restoration measures exhibited a gradual increase in ellipsoid biovolume and morphometric traits of individuals, indicating improved food availability and more favorable living conditions. Prediction of ellipsoid biovolume development using LSTM models (neural networks) indicated stabilization of Carabidae assemblages in forest habitats after the completion of restoration measures, whereas pronounced fluctuations persisted in open habitats, suggesting the need for a longer time period or more intensive interventions to achieve community stability. The results provide a practical basis for planning and evaluating the effectiveness of restoration and management measures in European habitats of conservation importance, as well as for biodiversity conservation and adaptive landscape management, particularly in optimizing interventions in forest and open ecosystems to achieve long-term stability of Carabidae assemblages.

## Introduction

The European-significant Dunajské luhy Landscape Protected Area represents one of the most ecologically valuable floodplain and wetland areas in Central Europe. It is characterized by a complex system of floodplain forests, wetlands, and alluvial meadows that are part of the Natura 2000 network and perform key functions in preserving biodiversity, ecosystem processes, and the hydrological connectivity of the Danube regional ecosystem (Laco, 2021). Natural inundations, a dynamic water regime, and the structural heterogeneity of habitats create suitable conditions with high diversity of plant and animal species, including ecologically important groups such as ground beetles (Carabidae), which are considered bioindicators of ecological stability, disturbance, and management interventions (Ludwiczak, Nietupski & Kosewska, 2020; Litavský et al., 2021; Makwela, Slotow & Munyai, 2023; Racoviceanu et al., 2023; Marchiori, Malheiros & Melo, 2025).

Historical interventions, including regulation of the Danube’s flow and fragmentation of floodplain habitats, have, however, significantly disrupted the ecological stability of this area (Funk et al., 2019). In response to these changes, extensive revitalization projects have been implemented in recent years, focusing on restoring the water regime, hydromorphological processes, and functional habitat connectivity (Jakubínský et al., 2021). Their effectiveness is often evaluated using bioindicator groups, in which Carabidae representing a suitable model taxon due to their sensitivity to microhabitat conditions, trophic changes, and habitat structure (Petrášová, Jarolímek & Medvecká, 2013). Their species composition, morphometric traits, and ecological requirements provide a detailed reflection of the state and dynamics of ecosystems (Rainio & Niemelä, 2003; Koivula, 2011). Given their varying mobility, habitat preferences, and responses to disturbances, they are a suitable model taxon for assessing the effectiveness of management interventions in both forest and non-forest ecosystems.

Habitat restoration is often accompanied by changes in vegetation, food availability, and microclimatic conditions, which are also reflected in Carabidae populations, particularly in the proportion of macropterous, brachypterous, and apterous species (Langraf et al., 2020). Changes in the representation of individual species make it possible to assess the degree of environmental disturbance and its subsequent regeneration. Previous research indicates that macropterous species are typical of disturbed and dynamically changing habitats, while brachypterous species prefer stable environments that have not been continuously disturbed over long periods (Shibuya et al., 2014; Rouabah et al., 2015).

The morphometric traits and ellipsoid biovolume of ground beetles (Coleoptera: Carabidae) represents a key tool for assessing ecological processes, environmental changes, and community responses to management interventions in the landscape. Morphometric traits such as body length, width, and height, along with individual body, reflect not only the physiological state of populations but also the availability of environmental resources, stress factors, and habitat quality (Lövei & Sunderland, 1996; Homburg et al., 2014; Papp et al., 2020; Costa et al., 2021). Several studies confirm that changes in habitat structure, including disturbances or revitalization measures, significantly influence the morphometric traits and ellipsoid biovolume of ground beetles (Sukhodolskaya, 2013; Pizzolotto, 2022). The importance of morphometric traits variables lies primarily in their ability to reflect food availability, microclimatic conditions, and species’ ecological requirements (Lövei & Sunderland, 1996; Ribera et al., 2001). For example, macropterous species tend to be typical of disturbed or dynamic habitats where higher mobility is required, whereas apterous and brachypterous species prefer stable, long-term undisturbed environments (Jelaska & Durbešič, 2009). These life strategies are closely linked to morphometric traits, ellipsoid biovolume and body proportions, which may vary significantly among different habitats depending on resource availability or the intensity of ecological changes (Blake et al., 1994).

In our study, we evaluate the impact of revitalization measures on the morphometry of the family Carabidae in the European-significant Dunajské luhy Landscape Protected Area. Subsequently, using a neural network model, we predict the development in the following years after the implementation of the revitalization measures.

## Materials and Methods

During the years 2020–2023, we conducted research on 15 study sites. The research took place in the Danube Floodplains Protected Landscape Area (CHKO Dunajské luhy), a region of European importance located in western Slovakia. The area covers 122.8 km^2^ and belongs to the Alpine Himalayan system and the Danubian Lowland. Climatically, the territory falls into a warm region with mild winters, where the average summer temperature is 25 °C and the average winter temperature is −3 °C. The soil is clay-loam, formed by the alluvium of the Danube River.

In the selected forest habitats (willow poplar floodplain forest, ash–alder floodplain forest, Pannonian poplar forest, reed communities of wetlands), the following revitalization measures were carried out: creation of new river branches in the Danube delta, and simulation of floods occurring twice a year (spring, summer).

In the open-habitat areas (pasture, poplar nursery, alluvial meadow, lowland hay meadow), the following revitalization measures were implemented: mowing of grass stands between the tree rows in the poplar nursery, carried out twice a year (spring, summer); annual mowing of meadow vegetation; and flooding of meadow areas once per year, cattle grazing.

**Figure 1:**
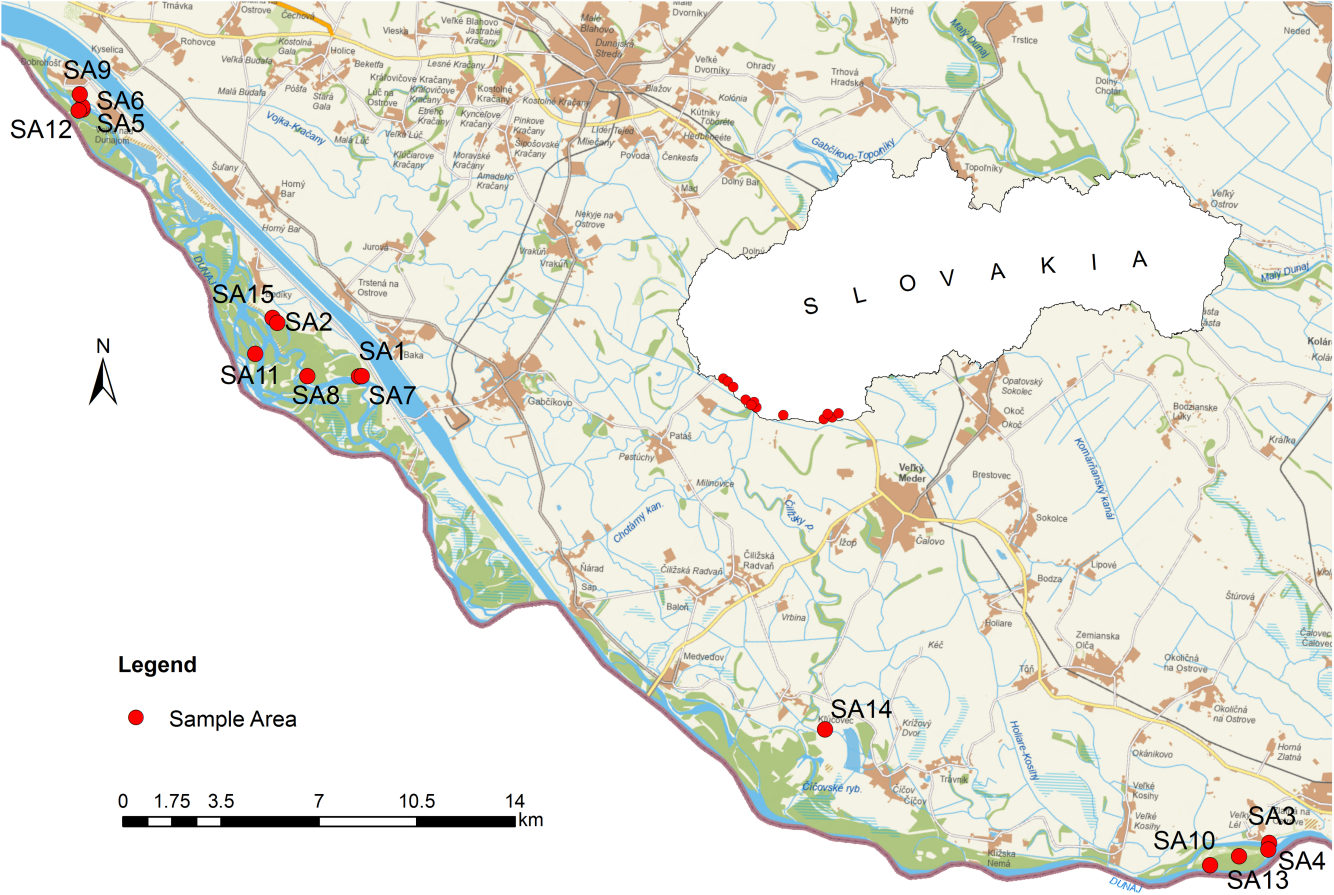
Map of study areas (1–15).

As control sites, we used two habitat types willow poplar floodplain forest and native pasture where no revitalization measures were applied. Study areas (1–15) are shown on the map (Fig. 1) and site characteristics are in Table 1.

**Table 1:** Characteristics study areas (1–15).

Study area	Geographic coordinates	m a.s.l.	Land-use type	Habitat
SA1	47°53′31.1″N, 17°30′25.4″E	114	Reference habitat	Willow–poplar floodplain forest
SA2	47°54′36.0″N, 17°27′51.3″E	119	Reference habitat	Willow–poplar floodplain forest
SA3	47°45′17.0″N, 17°56′59.9″E	106	Reference habitat	Willow–poplar floodplain forest
SA4	47°45′09.3″N, 17°56′58.8″E	108	Reference habitat	Pasture
SA5	47°58′24.5″N, 17°22′09.7″E	121	Revitalization measures	Ash–alder floodplain forest
SA6	47°58′27.4″N, 17°22′09.1″E	121	Revitalization measures	Pannonian poplar forest
SA7	47°53′31.5″N, 17°30′30.2″E	114	Revitalization measures	Willow–poplar floodplain forest
SA8	47°53′28.5″N, 17°28′56.9″E	115	Rrevitalization measures	Willow–poplar floodplain forest
SA9	47°58′41.6″N, 17°22′03.7″E	121	Revitalization measures	Reed communities of wetlands
SA10	47°44′48.3″N, 17°55′20.2″E	108	Revitalization measures	Poplar nursery
SA11	47°53′51.5″N, 17°27′25.5″E	118	Revitalization measures	Poplar nursery
SA12	47°58′22.9″N, 17°22′02.9″E	127	Revitalization measures	Poplar nursery
SA13	47°45′00.0″N, 17°56′09.2″E	108	Revitalization measures	Alluvial meadow
SA14	47°47′07.2″N, 17°44′11.4″E	110	Revitalization measures	Lowland hay meadow
SA15	47°54′33.8″N, 17°27′52.6″E	119	Revitalization measures	Pasture

**Note:**

m a.s.l. = elevation above sea level.

We collected ground beetles using pitfall traps at monthly intervals from April to October during the years 2020–2023. A 4% formalin solution was used as the fixing fluid. On each study site, five pitfall traps were placed in a line, with a distance of 10 m between each pitfall trap, resulting in a total line length of 40 m. In total, 75 pitfall traps were installed throughout the year. The ground beetles were identified using the key by Hůrka (1996). The statistical unit of the study is an individual beetle. For each captured individual, we measured morphometric traits (body length, body height, body width). Thus, each measured individual was considered an independent observation, and the values of morphometric traits for each individual were used in the statistical analysis.

### Ellipsoid biovolume of carabidae

Each collected individual was measured using a digital microscope, the Koolerton LCD microscope (model ADSM301, company: Shenzhen Andonstar Technology Co., country: China, year 2017) with an accuracy of 0.1 mm. For each individual, we measured the following morphometric traits:

(1) body length—dorsal length measured from the upper lip (labrum) to the terminal end of the elytra
(2) body height—maximum dorsoventral thickness measured on the left side of the body
(3) body width dorsal distance corresponding to the maximum width of the elytra

Subsequently, following Braun, Jones & Perner (2004), we calculated the ellipsoid biovolume (EV) for each individual based on these morphometric traits. The formula for calculating EV is as follows:

${\rm EV}_{{\rm i}=1} = (\pi/6) \times {\rm L} \times {\rm H} \times {\rm W}$

L = body length, H = body height, W = body width.

### Statistical analyses

We expressed the association of species with habitats with and without revitalization measures using Principal Component Analysis (PCA) in the program Canoco5 (Ter Braak & Šmilauer, 2012). We used multifactorial ANOVA to test for differences in the number of individuals between habitats and flight ability (apterous, brachypterous, macropterous). We also used it to test for differences in morphometric traits and EV between habitats and collection years (2020–2023). We used one-way ANOVA to test for differences in the number of individuals between habitats. We also used it to test for differences in morphometric traits and EV between habitats. We then performed a Tukey HSD *post-hoc* test. We identified homogeneous groups based on pairwise comparisons where habitats did not differ significantly (*p* > 0.05). Based on the Tukey HSD test, habitats were classified into homogeneous groups marked with letters (a–f). Habitats marked with the same letter do not form statistically significantly different groups, while different letters indicate significant differences. In the graphs, we then displayed the number of individuals, EV and morphometric traits on the Y axis. The habitats were displayed on the X axis. To predict the development of the Ellipsoid Biovolume, we applied a neural network, specifically a Long Short-Term Memory (LSTM) model. These analyses were performed in Python version 3.14 (2025), using the libraries pandas, numpy, matplotlib, sklearn and torch.

### Ethics approval committee

All aspects of trapping complied with EU Council Directive 2010/63 regarding the protection of animals used for experimental and other scientific purposes. All procedures performed in studies involving animals were in accordance with the ethical standards of the in-stitution or practice at which the studies were conducted. The research was approved by the Ethics Committee of the Constantine the Philosopher University in Nitra. The ethics committee form number is UKF-2023/1006-2:191013.

## Results

From the results of our research, we confirmed 5,292 individuals and 47 species with an average EV value per individual of 192 mm^3^. The highest average EV value was recorded in the habitats willow–poplar floodplain forest (280 mm^3^), alluvial meadow (273 mm^3^, with management measures), and willow–poplar floodplain forest (212 mm^3^, without management measures). The lowest average EV value was found in the habitats pasture (62 mm^3^, without management measures) and lowland hay meadow (72 mm^3^, with management measures). In the remaining habitats (with management measures), we recorded the following values: poplar nursery and Pannonian poplar forest 167 mm^3^, ash–alder floodplain forests 166 mm^3^, reed communities of wetlands 162 mm^3^, and pasture 106 mm^3^ (Table 2).

**Table 2:** EV values and number of individuals found in the investigated habitats.

Species	Flight ability	Without management measures		With management measures							
		A	B	C	D	E	F	G	H	I	J
		Ev/N	Ev/N	Ev/N	Ev/N	Ev/N	Ev/N	Ev/N	Ev/N	Ev/N	Ev/N
*Abax ovalis* (Duftschmid, 1812)	B	0/0	0/0	0/0	0/0	0/0	0/0	286/2	0/0	0/0	0/0
*Abax parallelepipedus* (Piller et Mitterpacher, 1783)	B	0/0	0/0	0/0	0/0	0/0	0/0	0/0	1,2817/31	0/0	0/0
*Abax parallelus* (Duftschmid, 1812)	B	0/0	0/0	0/0	0/0	208/1	6,194/38	298/2	1,217/8	0/0	0/0
*Amara aenea* (De Geer, 1774)	M	12/1	30/3	102/5	32/3	27/1	34/3	85/4	25/1	302/15	0/0
*Amara familiaris* (Duftschmid, 1812)	M	58/5	1,391/48	0/0	0/0	0/0	74/5	0/0	851/35	0/0	329/13
*Amara ingenua* (Duftschmid, 1812)	M	0/0	0/0	0/0	0/0	0/0	0/0	0/0	3,249/137	0/0	0/0
*Amara saphyrea* Dejean, 1828	M	71/2	61	76/2	693/16	232/6	137/4	0/0	44/1	0/0	0/0
*Anchomenus dorsalis* (Pontoppidan, 1763)	M	286/28	390/25	108/12	86/5	273/22	239/21	243/22	58/4	511/35	30/3
*Asaphidion austriacum* (Schweiger, 1975)	M	10/3	0/0	20/6	0/0	0/0	0/0	0/0	6/2	0/0	0/0
*Bembidion illigeri* (Netolitzky, 1914)	M	6/1	0/0	0/0	0/0	0/0	0/0	0/0	0/0	0/0	0/0
*Brachinus crepitans* (Linnaeus, 1758)	M	0/0	57/6	7/1	0/0	24/3	6/1	0/0	0/0	107/9	1,631/71
*Brachinus explodens* (Duftschmid, 1812)	M	0/0	88/3	55/2	0/0	0/0	0/0	0/0	23/1	195/10	84/4
Brachinus ganglbaueri Apfelbeck, 1904	M	0/0	0/0	0/0	0/0	0/0	0/0	0/0	0/0	260/12	0/0
*Calathus fuscipes* (Goeze, 1777)	M	812/5	7,254/100	7,065/82	0/0	7,183/82	332/5	0/0	17,298/251	3,133/37	10,372/154
*Calathus melanocephalus* (Linnaeus, 1758)	B	0/0	0/0	226/14	0/0	0/0	0/0	0/0	0/0	0/0	0/0
*Callistus lunatus* (Fabricius, 1775)	M	0/0	0/0	0/0	10/1	0/0	0/0	0/0	0/0	0/0	13/1
*Calosoma auropunctatum* (Herbst, 1784)	A	0/0	0/0	0/0	0/0	0/0	0/0	0/0	0/0	1,117/1	0/0
*Calosoma inquisitor* (Linnaeus, 1758)	M	0/0	0/0	0/0	0/0	996/2	0/0	0/0	0/0	0/0	0/0
*Carabus cancellatus* (Illiger, 1798)	A	760/1	0/0	0/0	0/0	0/0	0/0	0/0	312/1	0/0	0/0
*Carabus coriaceus* (Linnaeus, 1758)	A	26,414/12	0/0	0/0	2,898/7	14,784/6	10,105/4	7,180/3	14,090/5	0/0	0/0
*Carabus granulatus* (Linnaeus, 1758)	B	138,702/358	553/2	6,415/19	124,898/345	3,212/8	558/2	7,731/22	60,392/160	115,119/286	0/0
Carabus ullrichi (Germar, 1824)	A	3,867/4	0/0	0/0	0/0	1,921/2	0/0	6,287/6	4,477/5	864/1	0/0
*Cicindela campestris* (Linnaeus, 1758)	M	0/0	0/0	0/0	0/0	97/1	0/0	0/0	0/0	0/0	0/0
Cicindela germanica (Linnaeus, 1758)	M	0/0	0/0	565/14	0/0	0/0	0/0	0/0	0/0	83/2	300/7
*Cychrus caraboides* (Linnaeus, 1758)	A	1,694/5	0/0	0/0	6,354/19	0/0	0/0	550/2	788/2	0/0	0/0
*Drypta dentata* (Rossi, 1790)	M	0/0	0/0	32/2	0/0	0/0	0/0	0/0	36/2	24/1	0/0
*Elaphrus aureus* (P. Müller, 1821)	M	133/9	0/0	0/0	0/0	0/0	17/6	23/7	0/0	0/0	0/0
*Harpalus affinis* (Schrank, 1781)	M	147/4	119/2	129/3	0/0	0/0	0/0	0/0	35/1	0/0	130/4
*Harpalus caspius* roubali (Schauberger, 1928)	M	0/0	0/0	0/0	0/0	0/0	0/0	0/0	107/1	0/0	0/0
*Harpalus rubripes* (Duftschmid, 1812)	M	62/3	278/5	18/1	0/0	35/2	17/1	40/2	1,033/39	249/7	0/0
*Harpalus tardus* (Panzer, 1797)	M	210/4	28/1	0/0	0/0	0/0	579/12	0/0	84/2	0/0	0/0
*Chlaenius festivus* (Panzer, 1796)	M	0/0	0/0	0/0	0/0	0/0	0/0	3,265/22	181/1	0/0	317/1
*Chlaenius nigricornis* (Fabricius, 1787)	M	200/3	0/0	0/0	0/0	0/0	0/0	8,738/90	0/0	887/10	0/0
*Chlaenius vestitus* (Paykull, 1790)	M	0/0	0/0	0/0	0/0	0/0	0/0	40/1	0/0	0/0	0/0
*Leistus rufomarginatus* (Duftschmid, 1812)	M	26/1	0/0	0/0	0/0	0/0	0/0	0/0	0/0	0/0	0/0
*Nebria brevicollis* (Fabricius, 1792)	M	4,752/65	1,385/17	4,649/66	3,022/50	4,225/56	232/3	16,762/227	1,202/16	1,438/21	187/2
*Notiophilus biguttatus* (Fabricius, 1799)	B	15/3	0/0	6/1	15/3	6/1	10/2	11/6	0/0	0/0	0/0
*Ophonus azureus* (Fabricius, 1775)	B	0/0	0/0	42/1	0/0	0/0	0/0	0/0	0/0	0/0	0/0
*Platyderus rufus* (Duftschmid, 1812)	B	121/12	139/12	0/0	129/15	77/8	38/4	0/0	80/8	0/0	0/0
*Platynus assimilis* (Paykull, 1790)	M	26,738/462	58/1	54/1	2,028/18	0/0	0/0	232/4	0/0	514/7	0/0
*Poecilus cupreus* (Linnaeus, 1758)	M	0/0	35/1	3,503/14	0/0	0/0	0/0	0/0	0/0	59,580/188	82/1
*Poecilus versicolor* (Sturm, 1824)	M	3,227/39	310/4	67/1	0/0	0/0	0/0	429/5	332/6	1,647/18	1,945/22
Pseudoophonus rufipes (DeGeer, 1774)	M	470/4	3,048/21	1,109/8	213/2	273/3	0/0	104	12,229/88	1,213/10	6,364/53
*Pterostichus melanarius* (Illiger, 1798)	B	0/0	0/0	0/0	0/0	0/0	0/0	0/0	0/0	0/0	556/4
*Pterostichus niger* (Schaller, 1783)	M	49,334/180	704/3	5,683/27	54,668/213	659/2	0/0	37,259/120	14,165/61	12,026/61	3,549/18
*Pterostichus nigrita* (Paykull, 1790)	M	121/2	0/0	0/0	0/0	0/0	0/0	0/0	110/1	0/0	0/0
Trichocellus placidus (Gyllenhal, 1827)	M	6/1	0/0	0/0	0/0	0/0	0/0	7/1	0/0	0/0	0/0
**∑ EV/N**		**258,252/1,271**	**16,112/258**	**29,930/282**	**195,046/697**	**34,231/206**	**18,572/111**	**90,920/562**	**145,241/870**	**199,270/731**	**25,890/358**

**Note:**

Explanations: A, willow-poplar floodplain forest; B, pasture; C, pasture; D, willow-poplar floodplain forest; E, ash-alder floodplain forests; F, Pannonian poplar forest; G, Reed communities of wetlands; H, poplar nursery; I, alluvial meadow; J, Lowland hay meadow. Flight ability: A, apterous; B, brachypterous; M, macropterous.

Using principal component analysis (PCA) (gradient length = 1.9 on the first ordination axis), we examined the association of species with habitats where management measures were implemented, as well as with reference habitats without intervention. The values of the explained cumulative variability of the species data were 30.5% on the first ordination axis and 48.95% on the second ordination axis.

**Figure 2:**
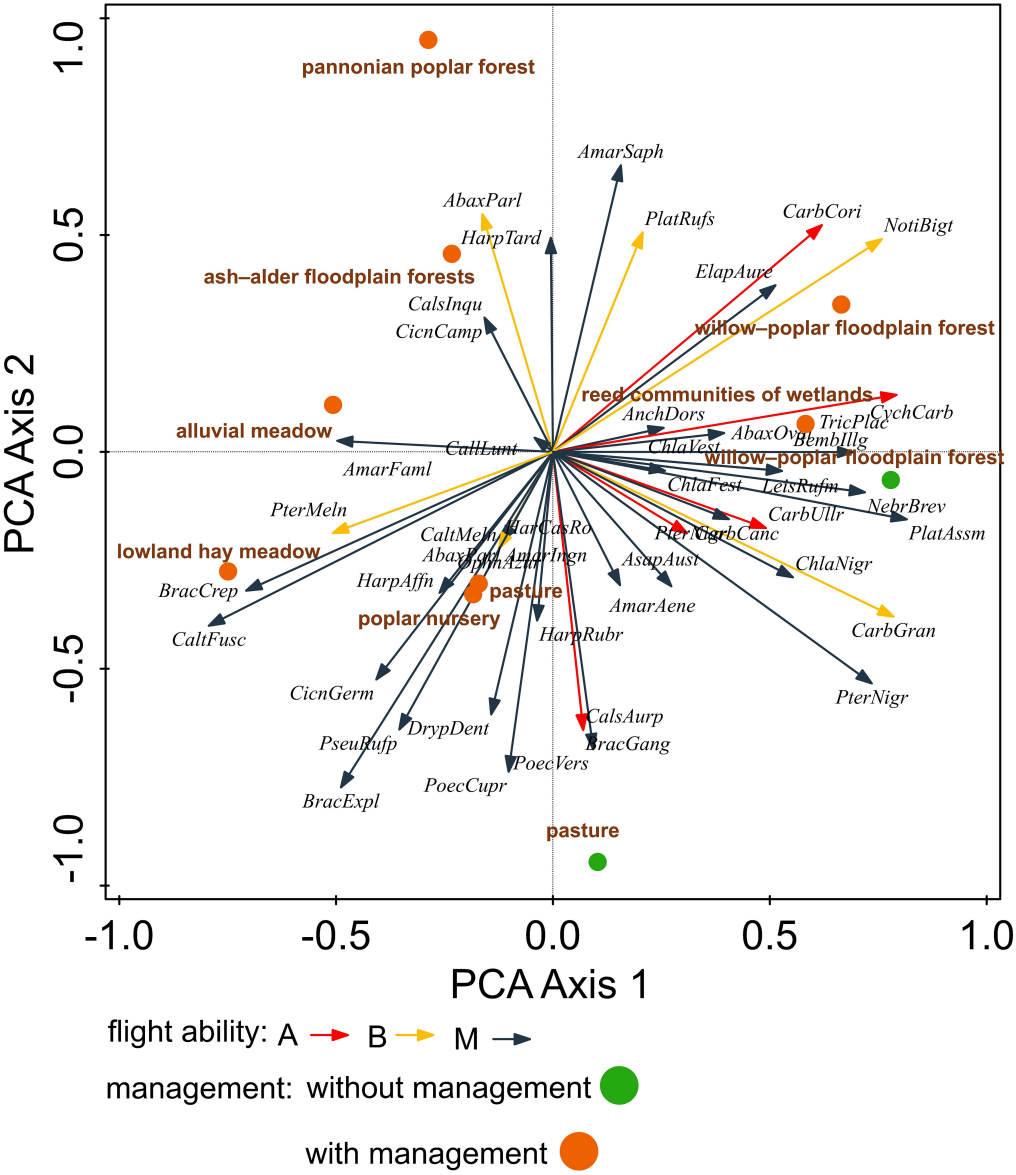
PCA analysis of the relationship of species (Carabidae) to habitats with and without management measures.

Based on the results, we can conclude that the species were divided into two main clusters. The first cluster includes species with a preference for forest habitats, while the second cluster comprises species correlated with open-habitat environments (meadows, pastures, poplar nursery). In the habitats that served as reference sites, where no revitalization measures were carried out, we recorded a predominance of apterous and brachypterous species compared to habitats where management interventions were implemented (Fig. 2).

**Figure 3:**
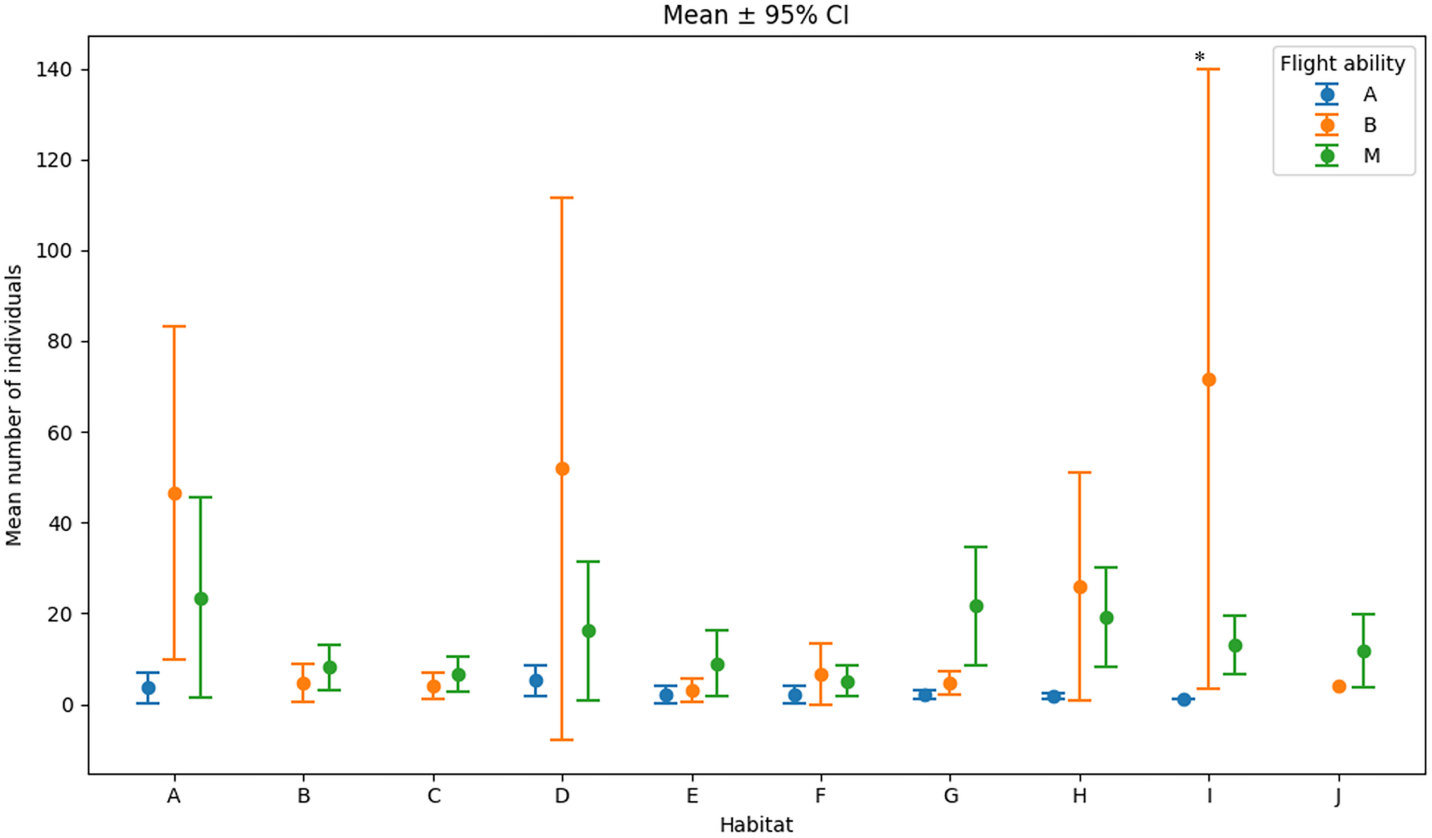
Difference in the number of individuals between habitats and flight ability. Explanations: A, willow-poplar floodplain forest; B, pasture (without management measures); C, pasture; D, willow-poplar floodplain forest; E, ash-alder floodplain forests; F, Pannonian poplar forest; G, Reed communities of wetlands; H, poplar nursery; I, alluvial meadow; J, Lowland hay meadow (with management measures).

A multifactor ANOVA was used to test differences in the number of individuals between habitats and flight ability (apterous, brachypterous, macropterous), which did not reveal a significant difference (*p* = 0.158). Using one-way ANOVA, we also did not confirm a significant difference in the number of individuals among habitats (*p* = 0.0845). Tukey test results indicate that differences are most pronounced between habitats with extreme values, while habitats with intermediate abundance tend to show partial overlap and fewer statistically significant contrasts (Fig. 3).

**Figure 4:**
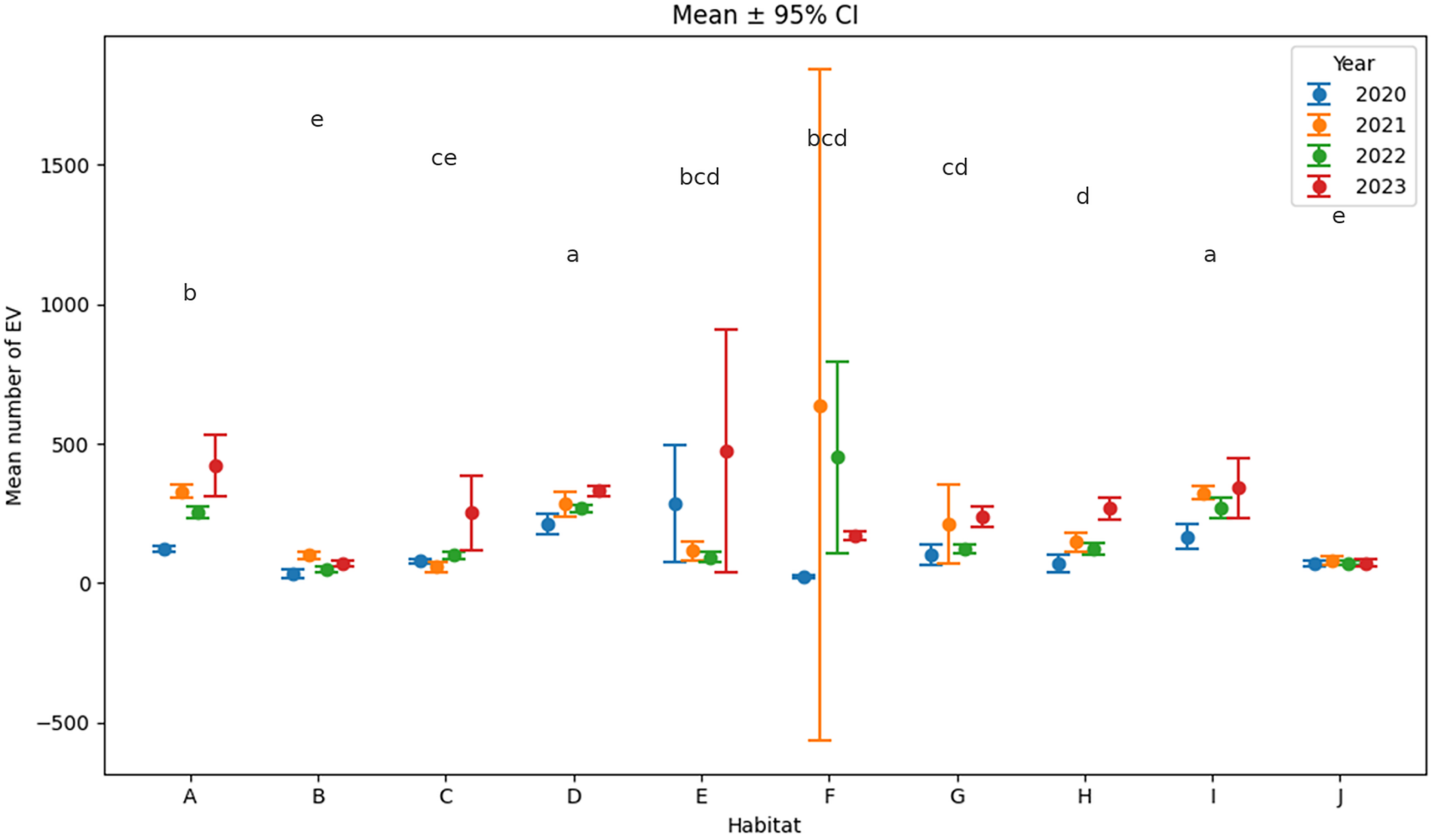
Difference in Ev values between habitats during 2020–2023. Explanations: A, willow-poplar floodplain forest; B, pasture (without management measures); C, pasture; D, willow-poplar floodplain forest; E, ash-alder floodplain forests; F, Pannonian poplar forest; G, Reed communities of wetlands; H, poplar nursery; I, alluvial meadow; J, Lowland hay meadow (with management measures).

**Figure 5:**
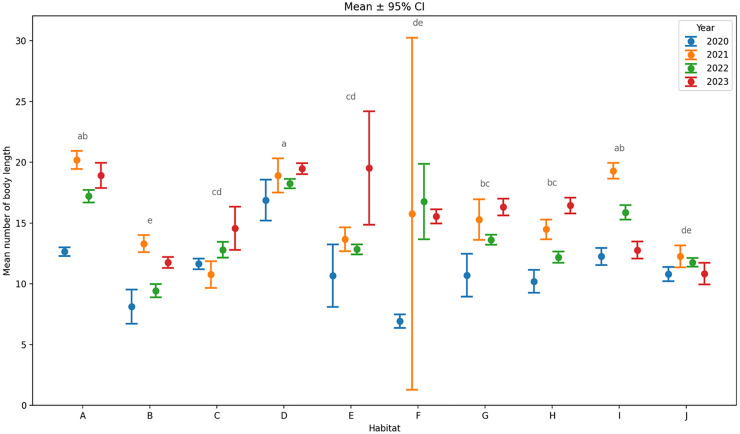
Difference in body length values between habitats during 2020–2023. Explanations: A, willow-poplar floodplain forest; B, pasture (without management measures); C, pasture; D, willow-poplar floodplain forest; E, ash-alder floodplain forests; F, Pannonian poplar forest; G, Reed communities of wetlands; H, poplar nursery; I, alluvial meadow; J, Lowland hay meadow (with management measures).

**Figure 6:**
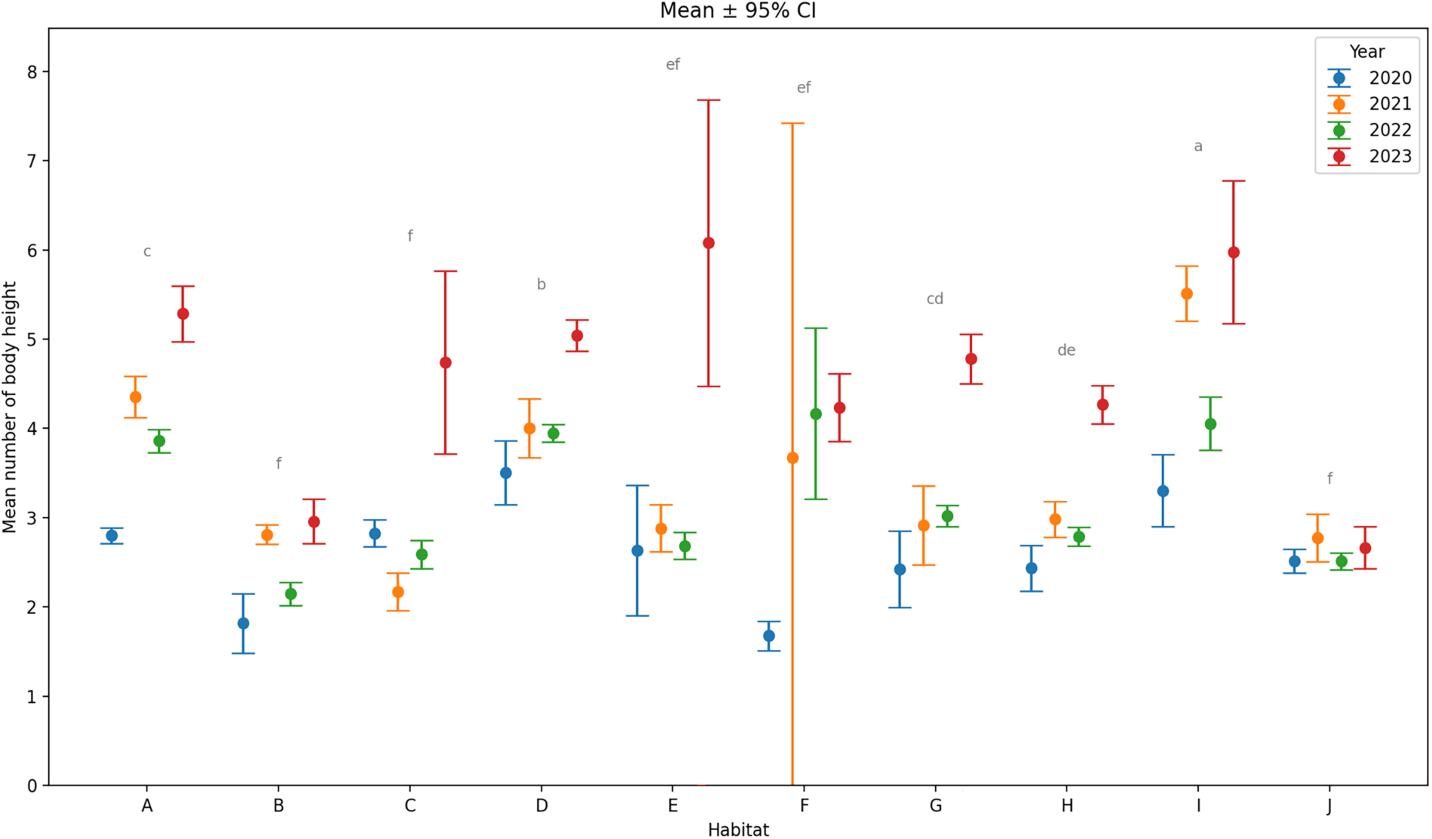
Difference in body height values between habitats during 2020–2023. Explanations: A, willow-poplar floodplain forest; B, pasture (without management measures); C, pasture; D, willow-poplar floodplain forest; E, ash-alder floodplain forests; F, Pannonian poplar forest; G, Reed communities of wetlands; H, poplar nursery; I, alluvial meadow; J, Lowland hay meadow (with management measures).

**Figure 7:**
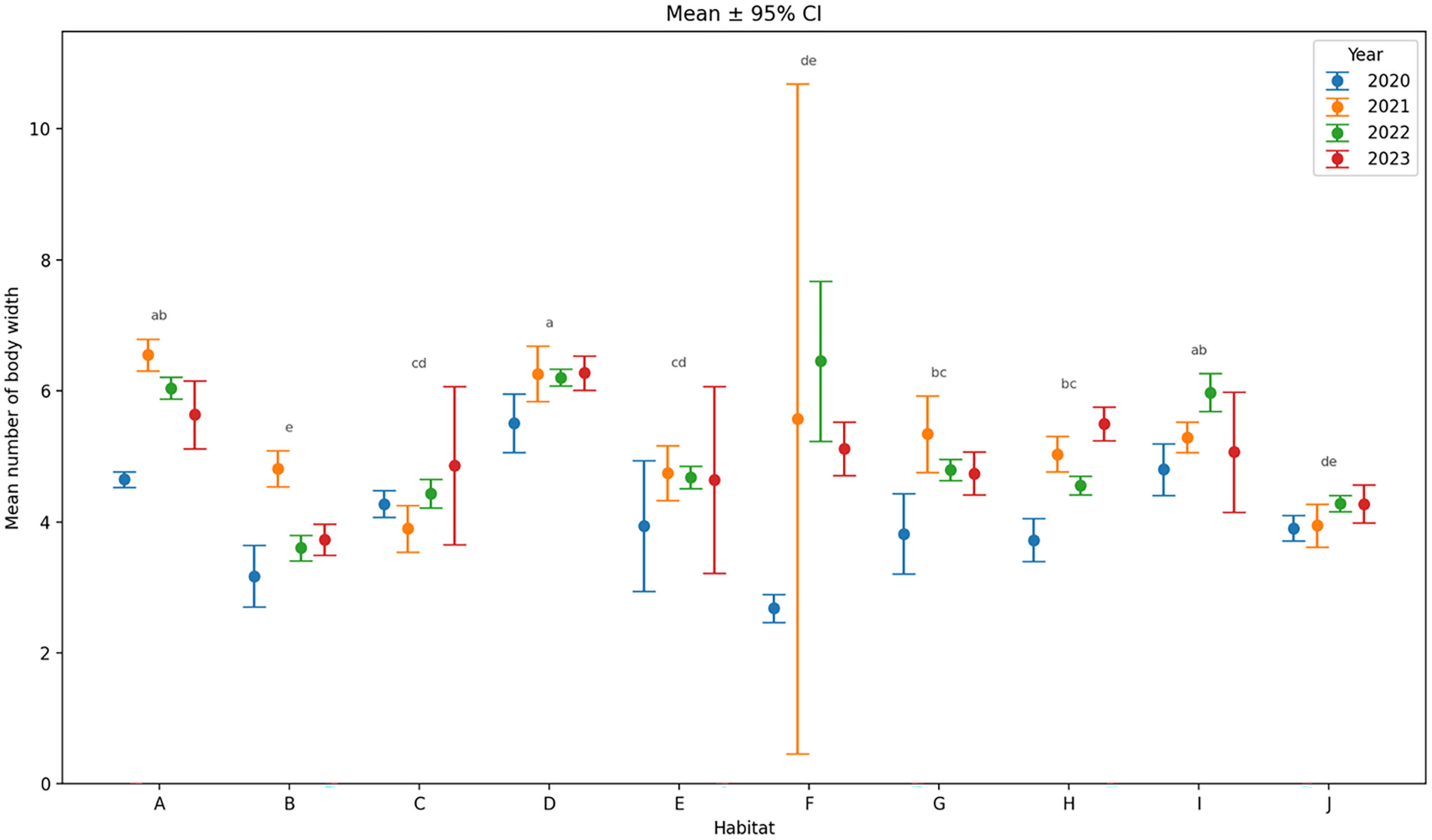
Difference in body width values between habitats during 2020–2023. Explanations: A, willow-poplar floodplain forest; B, pasture (without management measures); C, pasture; D, willow-poplar floodplain forest; E, ash-alder floodplain forests; F, Pannonian poplar forest; G, Reed communities of wetlands; H, poplar nursery; I, alluvial meadow; J, Lowland hay meadow (with management measures).

To test differences in EV and morphometric traits among habitats and years of sampling (2020–2023), a multifactor ANOVA was used. The results demonstrated a significant difference for EV (*p* = 0.00001) (Fig. 4) as well as for all morphometric traits: body length (*p* = 0.00001) (Fig. 5), body height (*p* = 0.000001) (Fig. 6), and body width (*p* = 0.000001) (Fig. 7). Additionally, one-way ANOVA was used to test differences EV and morphometric traits among habitats separately. The results confirmed significant differences for EV (*p* = 0.0001) and for all morphometric traits: body length (*p* = 0.0001), body height (*p* = 0.0001), and body width (*p* = 0.00001). Based on the results of the Tukey HSD test, statistically significant differences were found among the individual habitats in EV and morphometric traits (*p* < 0.05). The highest values were recorded in the habitats willow-poplar floodplain forest, alluvial meadow, and lowland hay meadow (with management measures), which differed significantly from habitats with the lowest values. Based on the morphometric traits and EV, we can conclude that in the forest habitat willow–poplar floodplain forest without the influence of revitalization measures, the values remained stable throughout all research years. In the forest habitats where revitalization measures were implemented—willow–poplar floodplain forest, ash–alder floodplain forests, Pannonian poplar forest, and reed communities of wetlands—we observed an increase in EV and morphometric traits. In the pasture where no revitalization measures were carried out, we confirmed stable values of morphometric traits and EV. However, we recorded a significant increase in the habitats pasture, poplar nursery, and alluvial meadow where revitalization measures were implemented. Between years, we observed a gradual increase in Ev and morphometric traits towards 2023.

**Figure 8:**
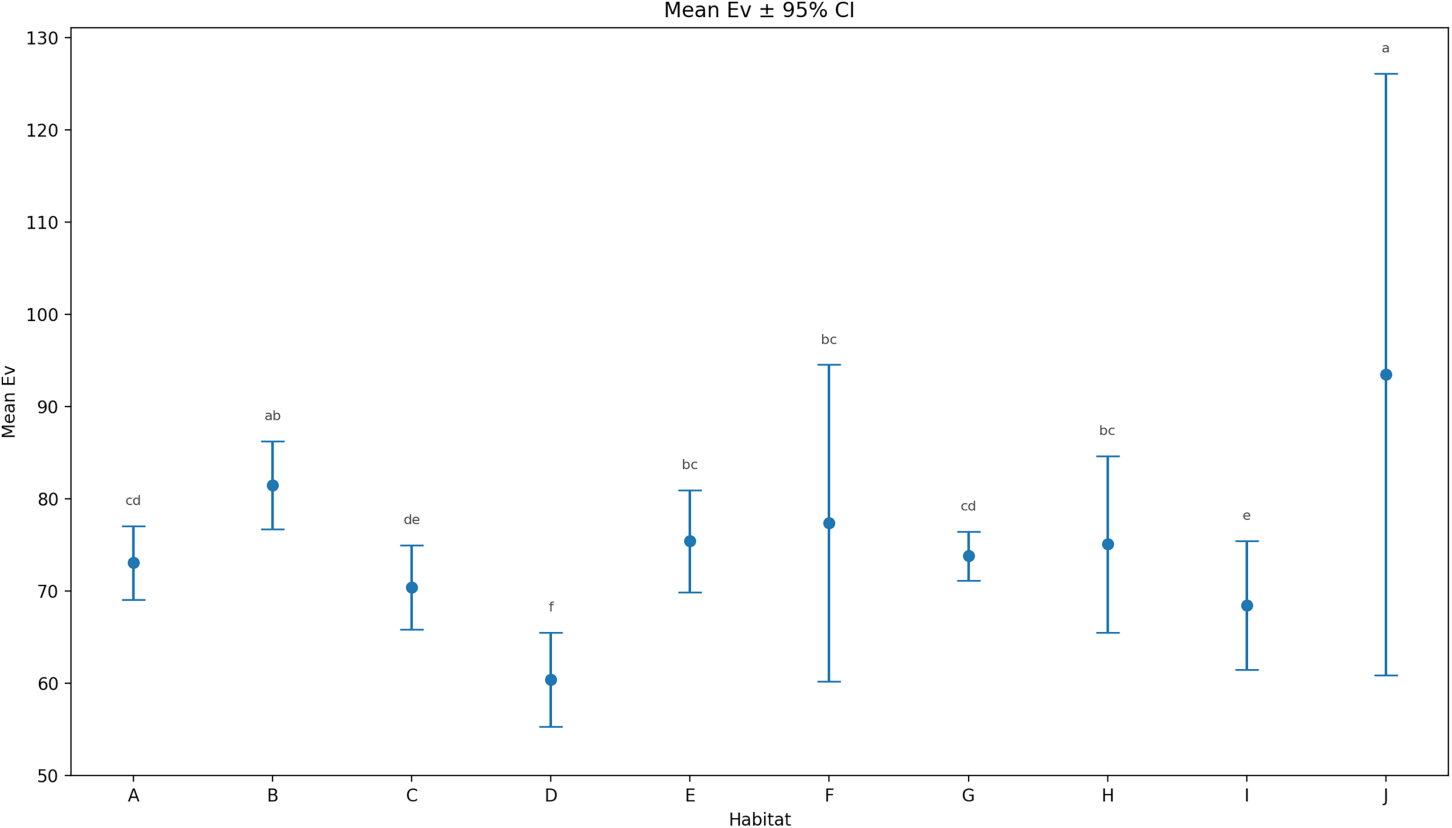
Differences in EV between habitats in a species *Nebria brevicollis*. Explanations: A, willow-poplar floodplain forest; B, pasture (without management measures); C, pasture; D, willow-poplar floodplain forest; E, ash-alder floodplain forests; F, Pannonian poplar forest; G, Reed communities of wetlands; H, poplar nursery; I, alluvial meadow; J, Lowland hay meadow (with management measures).

As a model species, we selected *Nebria brevicollis*, which was present in all studied habitats. Using one-way ANOVA, we tested the difference in EV among habitats and confirmed a significant difference (*p* = 0.0049). Based on the results of the Tukey HSD test, we can say that significant differences are present mainly between habitats with extreme values, while habitats with intermediate Ev values show partial overlap and fewer statistically significant differences. The highest mean EV value for this species was found in the habitat Lowland hay meadow and Pannonian poplar forest (with management measures). The lowest value was recorded in the willow-poplar floodplain forest habitat (with management measures) (Fig. 8).

Using a Long Short-Term Memory (LSTM) model, belonging to neural networks, we predicted the development of EV separately for forest habitats (willow–poplar floodplain forest, ash–alder floodplain forests, Pannonian poplar forest, reed communities of wetlands) where revitalization measures were implemented. The number of epochs during neural network training was set to 400. The Mean Absolute Percentage Error (MAPE), which expresses how much the model’s predictions differ on average from the actual values, was low—9.15%—indicating a very good model.

**Figure 9:**
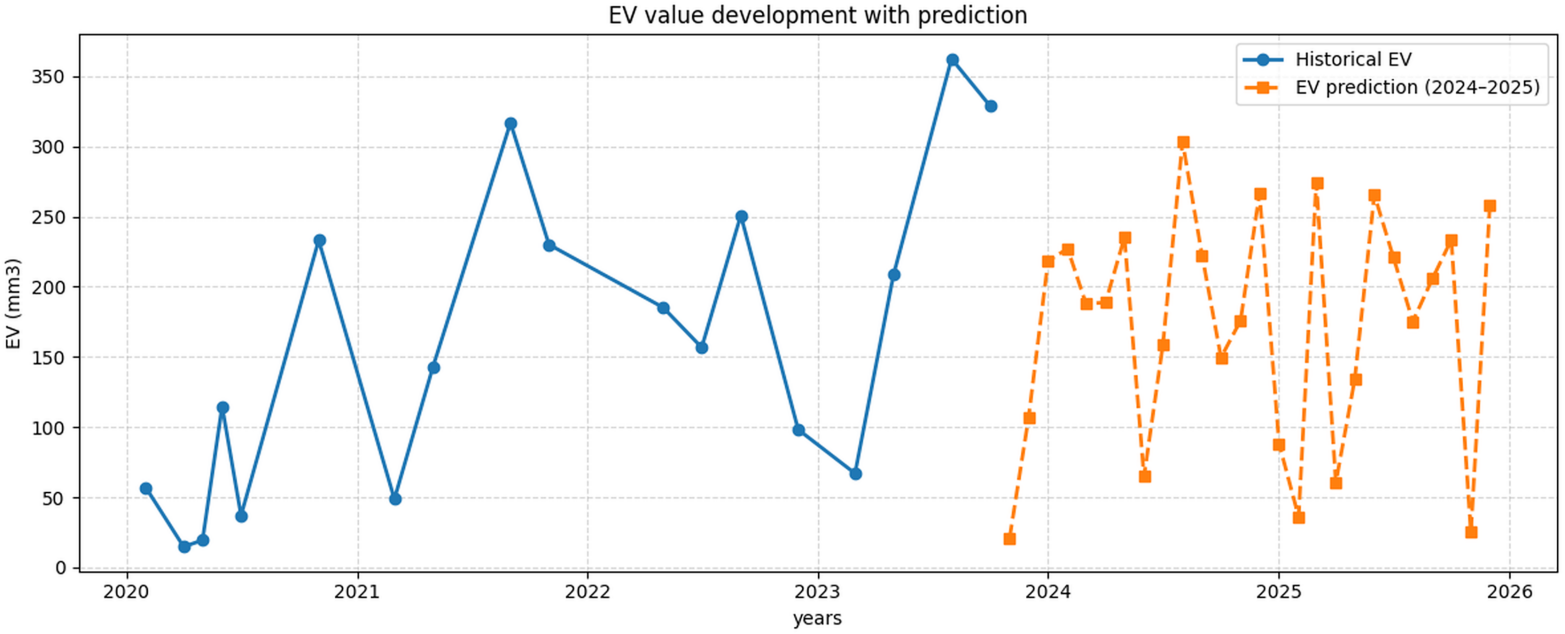
Long Short-Term Memory model of EV value development with prediction, on forest habitats with ongoing revitalization measures.

During the research period from 2020 to 2023, we observed fluctuations in the values, which are linked to the number of individuals and the availability of food—both influenced by the revitalization measures. An increase was recorded, becoming particularly pronounced at the end of 2023, when the effects of these measures peaked. The predicted model then shows the development for the years 2024 to 2025, where changes occur only due to seasonal variation, and the EV values remain stable without significant fluctuations (Fig. 9).

Subsequently, using a Long Short-Term Memory (LSTM) model belonging to neural networks, we predicted the development of EV separately for open-habitat environments (pasture, alluvial meadow, lowland hay meadow, poplar nursery) where revitalization measures were implemented. The number of epochs during neural network training was set to 500. The Mean Absolute Percentage Error (MAPE), expressing how much the model’s predictions differ on average from the actual values, was low—6.89%—which indicates a very good model.

**Figure 10:**
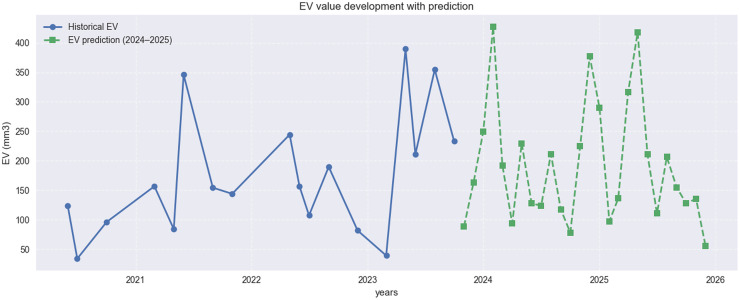
Long Short-Term Memory model of EV value development with prediction, on open space habitats with ongoing revitalization measures.

Throughout the 2020–2023 research period, the monitored values exhibited temporal variability, which can be attributed to changes in population density and resource availability driven by restoration activities. A pronounced upward trend was observed, culminating in late 2023 when the impact of the restoration measures was most evident. Projections for the 2024–2025 period indicate continued variability in the measured parameters; however, these fluctuations do not suggest the emergence of a consistent long-term directional trend (Fig. 10).

## Discussion

The research results demonstrated a significant effect of restoration measures on EV and morphometric traits of ground beetles, confirming their high sensitivity to environmental changes and food resource availability. The highest mean EV values were recorded in the willow–poplar floodplain forest and alluvial meadow habitats where restoration measures were implemented, indicating a positive effect of habitat structure recovery and increased food supply. Similar findings have been reported by other studies, which emphasize the importance of habitat and microhabitat heterogeneity for body size and overall condition of ground beetles (Magura, Lövei & Tóthmérész, 2010). In contrast, the lowest EV values were observed in unmanaged pastures, which corresponds to low structural diversity in intensively used or degraded habitats. These results are consistent with evidence that Carabidae respond to environmental stress by reducing body size and by increased energetic costs of movement under unstable conditions (Kotze et al., 2011).

Our results support the assumption that restoration measures influence not only species diversity but also the functional traits of ground beetle (Carabidae) populations. Increased structural heterogeneity of the environment and the availability of organic matter in restored habitats lead to more stable microclimatic conditions and a higher food supply, which is subsequently reflected in better body condition of individuals. According to Moraes, De Souza Mendonça & Ott (2013) and Bennewicz & Barczak (2020), habitat heterogeneity plays a key role in shaping the functional traits of ground beetles, with more complex environments supporting larger and physiologically more stable individuals. The relationship between EV values and habitat quality can also be explained by resource availability and the energetic strategies of organisms. Ludwiczak, Nietupski & Kosewska (2020) and Kaizuka, Yamaguchi & Iwasa (2020) point out that sufficient food availability and favorable environmental conditions allow individuals to allocate energy more efficiently to growth and reproduction, leading to higher body condition values and larger morphometric traits. In contrast, under stressful conditions, energy is redirected towards survival, which is reflected in a reduction of body size.

Principal Component Analysis revealed a clear differentiation of species assemblages according to habitat type and management regime. The clustering of species associated with forest and open habitats confirms the ecological specialization of ground beetles, with apterous and brachypterous species dominating in reference, stable habitats. These species prefer more stable habitats without cyclical changes or ecosystem disturbances. They have their nutritional optimum within the given habitat and do not need to disperse by flight to access food. The predominance of apterous and brachypterous species in unmanaged reference habitats suggests a well-established and stable ecological system, as these taxa are typically confined to environments characterized by long-term continuity and low disturbance. Their reduced dispersal capacity reflects an adaptation to stable conditions, where persistence rather than mobility is favored, and high dispersal ability provides no selective advantage (den Boer, 1990; Sota et al., 2022; Makwela, Slotow & Munyai, 2023). In contrast, the dominance of macropterous species in restored habitats reflects environmental disturbance and increased habitat dynamics, where higher mobility is advantageous for foraging and colonization of newly created microhabitats. These species colonizing disturbed ecosystems, where the food optimum is insufficient and individuals must fly to obtain food. This pattern is consistent with disturbance theory, which predicts that disturbed ecosystems favor species with higher dispersal capacity (Ribera et al., 2001). Furthermore, in forest habitats undergoing revitalization measures, we recorded a higher number of brachypterous species compared to meadow habitats with revitalization interventions. This indicates a higher effectiveness of revitalization measures in forest ecosystems. The highest mean number of individuals during the sampling years we recorded for brachypterous species in the habitats willow-poplar floodplain forest (with management measures), alluvial meadow, and willow-poplar floodplain forest (with management measures). The number of individuals of apterous species was relatively even across habitats. Macropterous species showed the highest number of individuals in the habitats willow-poplar floodplain forest (with management measures), willow-poplar floodplain forest, and reed communities of wetlands (with management measures). The occurrence of macropterous individuals in habitats supports the hypothesis that these forms are associated with more dynamic or disturbed environments due to their higher dispersal ability. In contrast, the relatively even distribution of apterous species across habitats may indicate their lower mobility and stronger association with stable microhabitats (Langraf et al., 2020). Thus, changes in morphometric traits and ellipsoid biovolume may also indicate a shift in species composition, where larger species of wingless and brachypterous beetles began to occur under stable conditions with optimal food availability.

The results of the morphometric analysis of the studied beetles across different habitat types over the period 2020–2023 indicate significant differences between sites with implemented restoration measures and habitats without direct management intervention. The results obtained clearly confirm statistically significant differences in EV as well as in all monitored morphometric traits. These findings are consistent with several studies highlighting the sensitivity of beetle body dimensions to environmental conditions, resource availability, and habitat quality (Sibly & Atkinson, 1994; Chown & Gaston, 2010). The EV represents an integrative parameter that incorporates multiple body size characteristics and is therefore a suitable indicator of overall individual condition. The increase in EV observed in restored habitats may result from higher food quality, improved availability of microhabitats, and reduced stress levels caused by environmental limiting factors. Which we also confirmed in the model species *Nebria brevicollis*, as well as in analyses of all species and individuals. According to energy allocation theory, organisms under favorable conditions allocate more energy to growth and body mass, whereas under stressful conditions energy is diverted toward survival and reproduction (Brygadyrenko & Reshetniak, 2014; Ludwiczak, Nietupski & Kosewska, 2020; Litavský et al., 2021; Makwela, Slotow & Munyai, 2023; Marchiori, Malheiros & Melo, 2025).

Particular attention should be given to the willow–poplar floodplain forest habitat without implemented restoration measures, where EV values as well as morphometric traits remained relatively stable throughout all monitored years. This stability indicates long-term established ecological conditions that allow beetle populations to maintain an equilibrium state (Sukhodolskaya et al., 2021). Stable body dimensions may result from a balanced food supply, consistent microclimatic conditions, and minimal disturbance, which is in accordance with the concept of ecological stability of natural or only slightly disturbed ecosystems (Brygadyrenko & Slynko, 2015). In contrast, forest and wetland habitats where restoration measures were applied—particularly willow–poplar floodplain forest, ash–alder floodplain forests, Pannonian poplar forest, and reed communities of wetlands—exhibited a pronounced increase in EV and in all morphometric parameters. This trend was especially evident in the final years of the study, culminating in 2023. Such a gradual increase in beetle body dimensions can be interpreted as a response to improving environmental conditions, primarily increased food availability, greater habitat structural diversity, and the restoration of the natural hydrological regime (Moraes, De Souza Mendonça & Ott, 2013).

An interesting aspect is the temporal dynamics of the observed changes, which indicate that the positive effects of restoration measures do not manifest immediately but develop gradually over time. This delayed response is consistent with principles of ecological succession, according to which the restoration of functional relationships within ecosystems occurs in several phases and may take several years (Walker & del Moral, 2003). The fact that the highest values of morphometric traits were recorded in 2023 points to a cumulative effect of management interventions. From a functional ecology perspective, increases in beetle body size may have important implications for the entire ecosystem. Larger individuals often exhibit higher food consumption, greater mobility, and different interactions with their environment, which can influence organic matter decomposition, regulation of other invertebrate populations, and energy flow within ecosystems (Loreau et al., 2001; Ludwiczak, Nietupski & Kosewska, 2020; Marchiori, Malheiros & Melo, 2025). Restoration measures therefore indirectly support functional stability and ecosystem resilience through changes in the morphology and condition of key invertebrate groups.

The application of neural network models in ecological research currently represents a significant methodological advance, particularly in the analysis and prediction of biological time series data. The LSTM model, which belongs to recurrent neural networks, is especially well suited for modeling population dynamics because it is capable of capturing long-term dependencies and nonlinear relationships in data (LeCun, Bengio & Hinton, 2015). In our study, the LSTM model was applied to predict the development of EV of Carabidae in habitats affected by restoration measures, with particular emphasis on differences between forest habitats and open-land habitats.

In forest habitats, the predictive model achieved high accuracy (MAPE = 9.15%). This result indicates that the development of Carabidae assemblages in these habitats is relatively stable and can be effectively captured using neural network models. Forest ecosystems are generally characterized by higher structural heterogeneity, a more stable microclimatic regime, and greater availability of shelters and food resources, which positively contributes to the stability of epigeic invertebrate communities (Niemelä et al., 2000; Gobbi et al., 2007). Fluctuations in EV values during the period 2020–2023 can be interpreted as a consequence of the gradual implementation of restoration measures, which influenced individual abundance and food availability. The pronounced increase in EV values toward the end of 2023 is likely associated with the culmination of restoration interventions and a delayed response of assemblages to improved environmental conditions. This pattern is consistent with ecological restoration theory, which suggests that the positive effects of management actions often manifest with a temporal lag (Suding, Gross & Houseman, 2004). Predictions of EV development for the years 2024–2025 indicate only seasonal fluctuations without pronounced long-term trends, suggesting stabilization of Carabidae assemblages following the completion of restoration measures. Such development supports the conclusion that the implemented interventions were sufficient in forest habitats and led to the restoration of ecological balance (Wu, He & Spence, 2020).

In contrast, in open-land habitats, pronounced variability in EV values persisted even after the completion of restoration measures, despite the high predictive accuracy of the model (MAPE = 6.89%). Open habitats are typically characterized by a higher degree of disturbance, more extreme microclimatic conditions, and more frequent human interventions, which may result in lower stability of soil invertebrate populations (Tscharntke et al., 2005; Porhajašová et al., 2018). The persistent fluctuations in EV observed in the predicted period of 2024–2025 suggest that Carabidae assemblages in these habitats have not yet reached a stable state and that the restoration measures were either insufficient or require a longer time horizon for their effects to fully manifest.

## Conclusions

Based on the data analyzed, it can be concluded that revitalization and management measures had a demonstrable impact on the structure and functional characteristics of ground beetle (Carabidae) communities in the studied habitats. The results showed that habitats where revitalization measures were applied achieved significantly higher mean values of Ellipsoid Biovolume as well as morphometric traits compared to reference habitats without intervention, indicating improved trophic conditions and overall life optimum for these organisms. There was also a change in species composition in habitats. The most pronounced positive effect was recorded in forest ecosystems, where not only an increase in EV was observed, but also a gradual stabilization of values over time, suggesting higher effectiveness of revitalization measures compared to open habitats. PCA analysis confirmed a clear differentiation between species associated with forest and open habitats and also highlighted different responses of species with distinct dispersal strategies to the degree of environmental disturbance. The prevailing occurrence of macropterous species and individuals in revitalized areas reflects the dynamics of disturbed ecosystems, whereas the dominance of brachypterous and apterous species and individuals in stable, unmanaged habitats indicates their dependence on long-term stable conditions. We confirmed significant differences in all monitored morphometric traits and Ellipsoid Biovolume among habitats and years of research, emphasizing the importance of management interventions over a longer time horizon. Predictive models based on neural networks demonstrated high accuracy and suggested that in forest habitats, Carabidae communities tend to stabilize after the completion of revitalization measures, whereas in open habitats higher variability persists and a longer period is required to achieve ecological balance. These findings confirm that the effectiveness of revitalization measures is strongly dependent on habitat type and intervention intensity, and that community responses are delayed, with the most pronounced positive effects becoming apparent only several years after their implementation. The practical application of these results lies in more targeted adjustment of management interventions in European habitats of conservation importance within the Dunajské luhy Protected Landscape Area. This applies particularly to forest ecosystems, where high effectiveness was confirmed, and highlights the need for longer-term and more intensive management of open habitats. Predictive models can serve as a tool for forecasting community development following interventions and for supporting decision-making in nature conservation, spatial planning, and sustainable landscape management.

## Supplemental Information

10.7717/peerj.21556/supp-1 Supplemental Information 1 Ellipsoid biovolume for each individual. Each collected individual was measured using a digital microscope, the Koolerton LCD microscope with an accuracy of 0.1 mm. For each individual, we measured the following morphometric traits: 1) body length–dorsal length measured from the upper lip (labrum) to the terminal end of the elytra; 2) body height–maximum dorsoventral thickness measured on the left side of the body; 3) body width dorsal distance corresponding to the maximum width of the elytra; Subsequently, following Braun et al. (2004), we calculated the Ellipsoid Biovolume (EV) for each individual based on these morphometric traits.

10.7717/peerj.21556/supp-2 Supplemental Information 2 Habitat Willow–poplar floodplain forest (reference habitat)—Study area 1. (SA1) = willow–poplar floodplain forest (reference habitat where no revitalization measures were carried out). (Figure 11). Botanical description: The tree layer is formed by the species *Salix fragilis* Linnaeus (1753) and *Salix alba*. Crawford, (1914) The herbaceous layer was represented by species *Urtica dioica* Linné, 1753, *Impatiens glandulifera* Royle. (1834), *Solidago gigantea* Aiton. (1789), *Galium odoratum* Fl. Carniol (1771), *Parietaria officinalis* Linnaeus (1753), *Rubus caesius* Linnaeus (1753), *Stachys sylvatica* Linnaeus (1753), and *Lamium maculatum* Linnaeus (1763).

10.7717/peerj.21556/supp-3 Supplemental Information 3 Habitat Willow–poplar floodplain forest (reference habitat)—Study area 2. (SA2) = willow–poplar floodplain forest (reference habitat where no revitalization measures were carried out) ( Figure 12). Botanical description: The tree layer is formed by the species *S. alba*, *S. fragilis*, *Populus × canadensis* Moench (1785) , *Acer negundo* Linnaeus (1753) , *Crataegus monogyna* Jacquin (1775) and *Populus × canescens* Smith (1804). The shrub layer consists of *Sambucus nigra* Linnaeus (1753) , *A. negundo* and *Swida sanguinea* Opiz (1852). The herb layer was represented by the species *I. glandulifera*, *U. dioica*, *R. caesius*, *Valeriana officinalis* Linnaeus (1753) , *Arctium nemorosum* Lejeune (1833), *Impatiens parviflora* de Candolle (1824) , *Roegneria canina* Nevski (1933), and *Galium aparine* Linnaeus (1753).

10.7717/peerj.21556/supp-4 Supplemental Information 4 Habitat Willow–poplar floodplain forest (reference habitat)—Study area 3. (SA3) = willow–poplar floodplain forest (reference habitat where no revitalization measures were carried out) ( Figure 13). Botanical description: The tree layer is composed of *S. alba*, *S. fragilis*, *Populus alba* Linnaeus (1753) , *P. × canescens*, *P. nigra*, *Alnus glutinosa* Gaertn (1790) , and *A. negundo*. The shrub layer consists of *S. nigra*, *Fraxinus excelsior* Linnaeus (1753) , *A. negundo*, *S. sanguinea*, and *Corylus avellana* Linnaeus (1753). The herb layer was represented by the species *U. dioica*, *Phalaroides arundinacea* Rauschert 1963, *S. gigantea*, *Aster lanceolatus* Nuttall (1818), *R. caesius*, and *Humulus lupulus* Linnaeus (1753).

10.7717/peerj.21556/supp-5 Supplemental Information 5 Habitat Pasture (original grassland, reference habitat)—Study area 4. (SA4) = pasture (original grassland, reference habitat where no revitalization measures were carried out) ( Figure 14). Botanical description: The tree layer consists of solitary individuals of *S. alba*. The herb layer was represented by the species *U. dioica*, *Cirsium arvense* Scopoli (1771), *Eryngium campestre* Linnaeus (1753) , *Achillea millefolium* Linnaeus (1753) , *Crepis biennis* Linnaeus (1753) , *Plantago lanceolatum* Linnaeus (1753) , *P. major*, *Rumex crispus* Linnaeus (1753) , *Elytrigia repens* Nevski (1933), *Dactylis glomerata* Linnaeus (1753) , *Arrhenatherum elatius* Presl & Presl (1819), *Cichorium intybus* Linnaeus (1753) , *Carduus acanthoides* Linnaeus (1753) , *Trifolium repens* Linnaeus (1753) , *Ranunculus repens* Linnaeus (1753) , *Euphorbia palustris* Linnaeus (1753) , *Eryngium planum* Linnaeus (1753) , *Centaurea jacea* Linnaeus (1753) , and *Setaria pumila* Roemer & Schultes (1817).

10.7717/peerj.21556/supp-6 Supplemental Information 6 Habitat Pannonian poplar forest—Study area 6. (SA6) = Pannonian poplar forest (forest habitat where revitalization measures were carried out = e xpansion of the branches of the Danube delta on the biotope, simulated flooding) ( Figure 16). Botanical description: The tree layer is formed by *Populus nigra* Linnaeus (1753) , *P. alba*, and *S. alba*. The herb layer was represented by the species *U. dioica*, *R. caesius*, *G. aparine*, *R. canina*, *S. gigantea*, *D. glomerata*, *E. repens*, and *Stenactis annua* Nees (1832).

10.7717/peerj.21556/supp-7 Supplemental Information 7 Habitat Willow–poplar floodplain forest—Study area 7. (SA7) = willow–poplar floodplain forest (forest habitat where revitalization measures were carried out = e xpansion of the branches of the Danube delta on the biotope, simulated flooding) ( Figure 17). Botanical description: The tree layer is composed of *S. fragilis* and *S. alba*. The herb layer was represented by the species *U. dioica*, *I. glandulifera*, *S. gigantea*, *G. odoratum*, *P. officinalis*, *R. caesius*, *S. sylvatica*, and *L. maculatum*.

10.7717/peerj.21556/supp-8 Supplemental Information 8 Habitat Willow–poplar floodplain forest—Study area 8. (SA8) = willow–poplar floodplain forest (forest habitat where revitalization measures were carried out = e xpansion of the branches of the Danube delta on the biotope, simulated flooding) ( Figure 18). Botanical description: The tree layer is composed of *P. × canadensis*, *P. robusta*, *S. fragilis* and *Viscum album* Linnaeus (1753). The shrub layer consists of *S. nigra*, *Cornus sanguinea* Linnaeus (1753) , and *A. negundo*. The herb layer was represented by the species *Phragmites australis* Steudel (1841), *U. dioica*, *G. aparine*, *Ficaria bulbifera* Holub (1961), *Carduus crispus* Linnaeus (1753) , *Symphyotrichum lanceolatum* Nesom (1995), *R. caesius*, *I. glandulifera*, *Sparganium erectum* agg. Linnaeus (1753), *Arctium* sp. Linnaeus (1753), *H. lupulus*, *Galeopsis speciosa* Miller (1768) and *Cucubalus baccifer* Linnaeus (1753).

10.7717/peerj.21556/supp-9 Supplemental Information 9 Habitat Reed communities of wetlands—Study area 9. (SA9) = Reed communities of wetlands (the edge of a wetland directly connected to a forest habitat, where revitalization measures were carried out = e xpansion of the branches of the Danube delta on the biotope, simulated flooding) ( Figure 19). Botanical description: The tree layer is composed of *S. alba*, *P. × canadensis*, and *A. negundo*. The herb layer was represented by the species *P. australis*, *U. dioica*, *Lythrum salicaria* Linnaeus (1753), *R. caesius*, and *S. gigantea*.

10.7717/peerj.21556/supp-10 Supplemental Information 10 Habitat Poplar nursery—Study area 10. (SA10) = poplar nursery (planted poplar nursery where revitalization measures were carried out = e xpansion of the branches of the Danube delta on the biotope, simulated flooding) ( Figure 20). Botanical description: The study area was planted with *P. alba* and *P. × canescens*. It is a 2-year-old stand without a tree or shrub layer. The herb layer was represented by the species *R. caesius*, *S. gigantea*, *P. alba*, *S. sanguinea* agg., *A. lanceolatus*, *U. dioica*, *Chenopodium album* Linnaeus (1753), *Symphytum officinale* Linnaeus (1753), *Stellaria media* Villars (1789), *Geum urbanum* Linnaeus (1753), *Erigeron annuus* Persoon (1807) and *E. repens*.

10.7717/peerj.21556/supp-11 Supplemental Information 11 Habitat Poplar nursery—Study area 11. (SA11) = poplar nursery (planted poplar nursery where revitalization measures were carried out = e xpansion of the branches of the Danube delta on the biotope, simulated flooding) ( Figure 21). Botanical description: The study area was planted with *P. alba* and *P. × canescens*. It is a 2-year-old stand without a tree or shrub layer. The herb layer was represented by the species *R. caesius*, *S. gigantea*, *P. alba*, *S. sanguinea* agg., *A. lanceolatus*, *U. dioica*, *Ch. album*, *S. officinale*, *S. media*, *G. urbanum*, *E. annuus* and *E. repens*.

10.7717/peerj.21556/supp-12 Supplemental Information 12 Habitat Poplar nursery—Study area 12. (SA12) = poplar nursery (planted poplar nursery where revitalization measures were carried out = e xpansion of the branches of the Danube delta on the biotope, simulated flooding) ( Figure 22). Botanical description: The study area was planted with *P. alba* and *P. × canescens*. It is a 2-year-old stand without a tree or shrub layer. The herb layer was represented by the species *R. caesius*, *S. gigantea*, *P. alba*, *S. sanguinea* agg., *A. lanceolatus*, *U. dioica*, *Ch. album*, *S. officinale*, *S. media*, *G. urbanum*, *E. annuus* and *E. repens*.

10.7717/peerj.21556/supp-13 Supplemental Information 13 Habitat Alluvial meadow—Study area 13. (SA13) = alluvial meadow (meadow habitat where revitalization measures were carried out = e xpansion of the branches of the Danube delta on the biotope, simulated flooding) ( Figure 23). Botanical description: Solitary individuals of *Taraxacum* sec. *Ruderalia* occurred within the study area. The herb layer was represented by the species *A. millefolium*, *C. biennis*, *P. lanceolatum*, *P. major*, *E. repens*, *D. glomerata*, *A. elatius*, *C. intybus*, *T. repens*, *C. jacea*, *S. pumila*, *R. caesius*, *S. officinale*, *R. repens*, *R. acris*, *Leontodon autumnalis* Oeder (1816), *Thlaspi perfoliatum* Linnaeus (1753), *R. crispus*, *E. palustris*, *Iris pseudacorus* Linnaeus (1753), and *Galium boreale* Linnaeus (1753).

10.7717/peerj.21556/supp-14 Supplemental Information 14 Habitat Lowland hay meadow—Study area 14. (SA14) = Lowland hay meadow (meadow habitat where revitalization measures were carried out = e xpansion of the branches of the Danube delta on the biotope, simulated flooding) ( Figure 24). Botanical description: The herb layer on the study area was represented by the species *E. annuus*, *D. glomerata*, *C. intybus*, *Plantago media* Linnaeus (1753), *C. arvense*, *A. millefolium*, *E. repens*, *S. gigantea*, *Inula britannica* Bieberstein (1808), and *S. officinale*.

10.7717/peerj.21556/supp-15 Supplemental Information 15 Habitat Pasture—Study area 15. (SA15) = pasture (grassland where revitalization measures were carried out = expansion of the branches of the Danube delta on the biotope, simulated flooding, grazing by cattle) ( Figure 25). Botanical description: Solitary individuals of *S. alba* occurred within the study area. The herb layer was represented by the species *C. arvense*, *C. acanthoides*, *A. lanceolatus*, *Artemisia campestris* Linnaeus (1753), *U. dioica*, *R. caesius*, *Agropyron repens* Beauvois (1812), *Lactuca serriola* Linnaeus (1756), *Tanacetum vulgare* Linnaeus (1753), *Glechoma hederacea* Linnaeus (1753), *Stenactis annua* Nees (1832), *A. millefolium*, *Poa pratensis* Linnaeus (1753), *P. media*, *Potentilla reptans* Linnaeus (1753), *Picris hieracioides* Linnaeus (1753), *G. parviflora*, and *Verbena officinalis* Linnaeus (1753).

## Additional Information and Declarations

### Field Study Permissions

The following information was supplied relating to field study approvals (*i.e*., approving body and any reference numbers):

Permission to access the Danube Floodplains Protected Landscape Area (CHKO Dunajské luhy) was provided by the ODBOR STAROSTLIVOSTI O ŽIVOTNÉ PROSTREDIE of the OKRESNÝ ÚRAD NITRA (OU-NR-OSZI1-2017/006679, OU-NR-OSZP1-2022/050775-002, OU-NR-OSZP1-2024/008666-002).

### Data Availability

The following information was supplied regarding data availability:

The raw data is available in the Supplemental Files.
