# Supplementary figures and images for: Morphometric variability of Carabidae as an indicator of ecological restoration of revitalized habitats in the European important protected landscape area of the Danube floodplains
Source: PeerJ. 2026 Jul 29;14:e21556. doi: 10.7717/peerj.21556 (PMC13428545; doi:10.7717/peerj.21556)

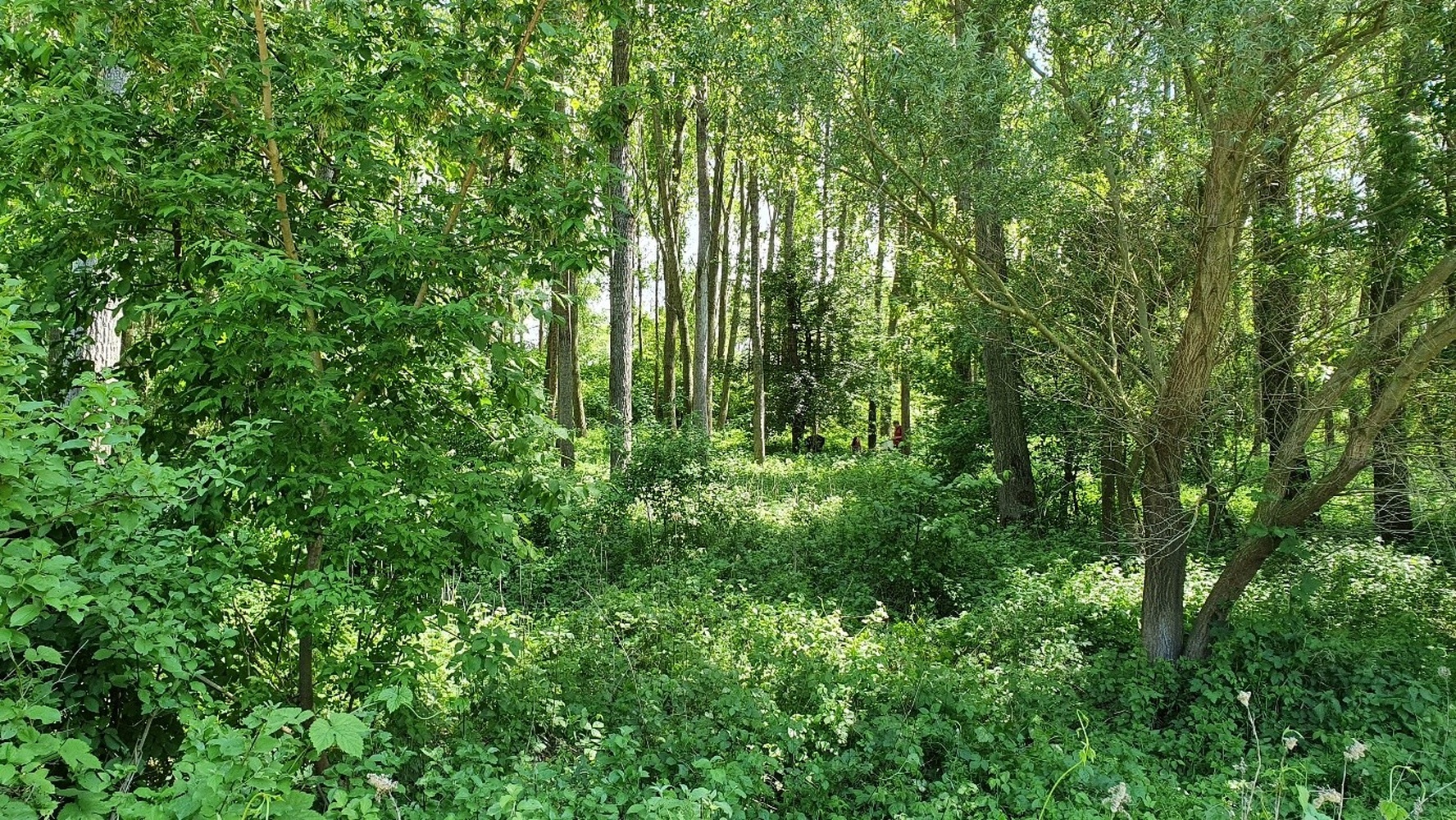

Supplement: Supplemental Information 2 — (SA1) = willow–poplar floodplain forest (reference habitat where no revitalization measures were carried out). (Figure 11). Botanical description: The tree layer is formed by the species Salix fragilis Linnaeus (1753) and Salix alba. Crawford, (1914) The herbaceous layer was represented by species Urtica dioica Linné, 1753, Impatiens glandulifera Royle. (1834), Solidago gigantea Aiton. (1789), Galium odoratum Fl. Carniol (1771), Parietaria officinalis Linnaeus (1753), Rubus caesius Linnaeus (1753), Stachys sylvatica Linnaeus (1753), and Lamium maculatum Linnaeus (1763). [file peerj-14-21556-s002.jpg]

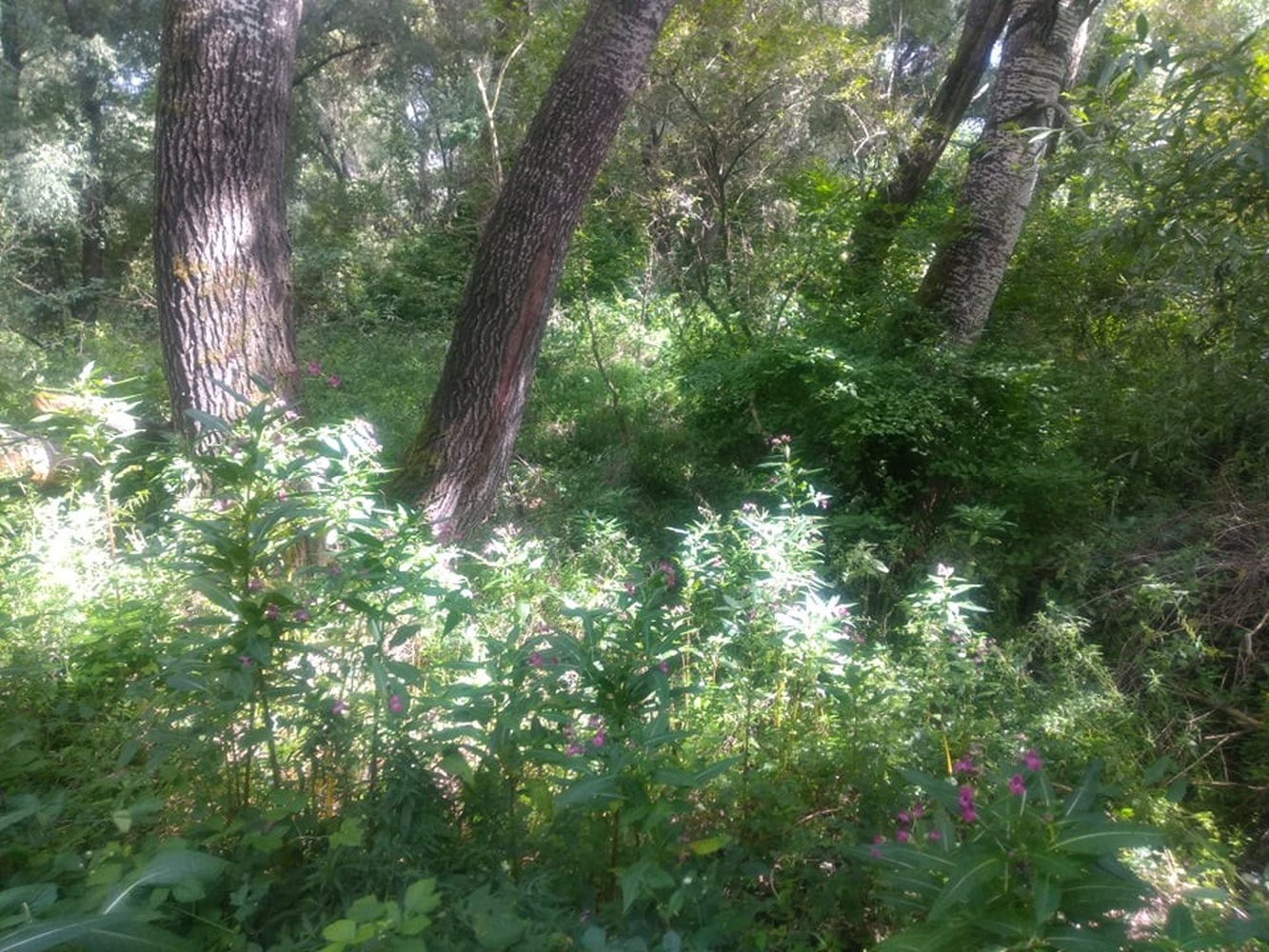

Supplement: Supplemental Information 3 — (SA2) = willow–poplar floodplain forest (reference habitat where no revitalization measures were carried out) ( Figure 12). Botanical description: The tree layer is formed by the species S. alba, S. fragilis, Populus × canadensis Moench (1785) , Acer negundo Linnaeus (1753) , Crataegus monogyna Jacquin (1775) and Populus × canescens Smith (1804). The shrub layer consists of Sambucus nigra Linnaeus (1753) , A. negundo and Swida sanguinea Opiz (1852). The herb layer was represented by the species I. glandulifera, U. dioica, R. caesius, Valeriana officinalis Linnaeus (1753) , Arctium nemorosum Lejeune (1833), Impatiens parviflora de Candolle (1824) , Roegneria canina Nevski (1933), and Galium aparine Linnaeus (1753). [file peerj-14-21556-s003.jpg]

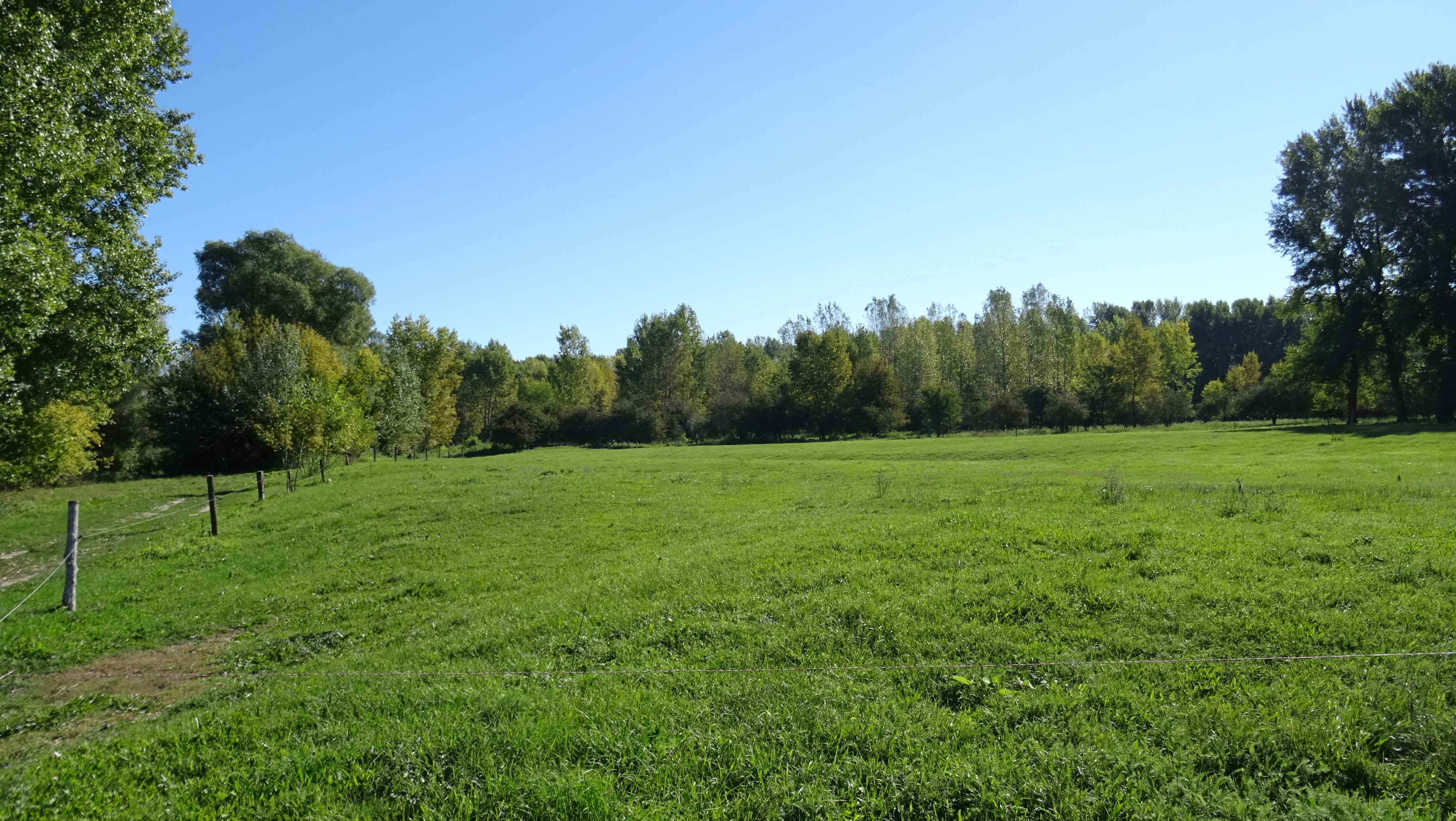

Supplement: Supplemental Information 4 — (SA3) = willow–poplar floodplain forest (reference habitat where no revitalization measures were carried out) ( Figure 13). Botanical description: The tree layer is composed of S. alba, S. fragilis, Populus alba Linnaeus (1753) , P. × canescens, P. nigra, Alnus glutinosa Gaertn (1790) , and A. negundo. The shrub layer consists of S. nigra, Fraxinus excelsior Linnaeus (1753) , A. negundo, S. sanguinea, and Corylus avellana Linnaeus (1753). The herb layer was represented by the species U. dioica, Phalaroides arundinacea Rauschert 1963, S. gigantea, Aster lanceolatus Nuttall (1818), R. caesius, and Humulus lupulus Linnaeus (1753). [file peerj-14-21556-s004.jpg]

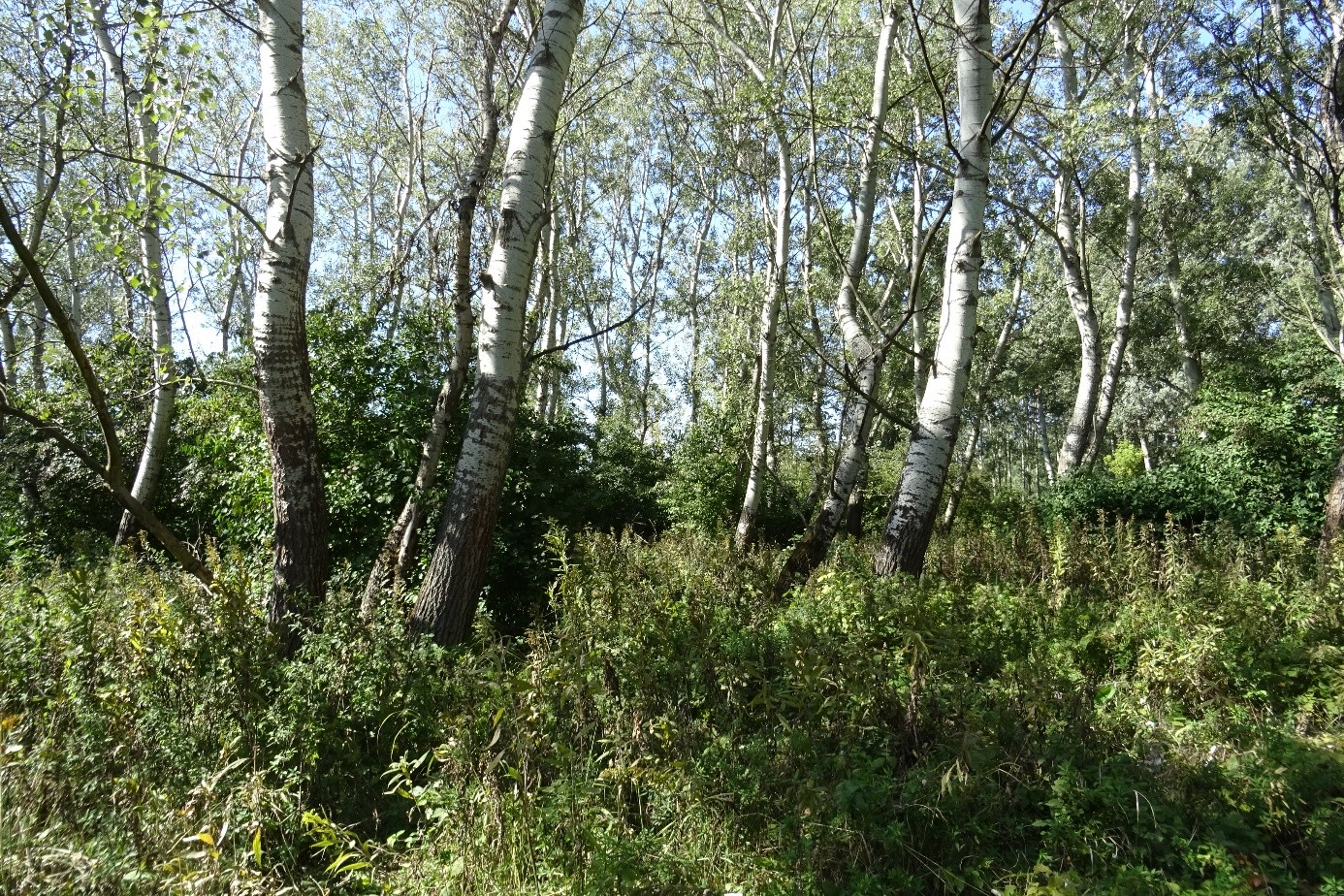

Supplement: Supplemental Information 5 — (SA4) = pasture (original grassland, reference habitat where no revitalization measures were carried out) ( Figure 14). Botanical description: The tree layer consists of solitary individuals of S. alba. The herb layer was represented by the species U. dioica, Cirsium arvense Scopoli (1771), Eryngium campestre Linnaeus (1753) , Achillea millefolium Linnaeus (1753) , Crepis biennis Linnaeus (1753) , Plantago lanceolatum Linnaeus (1753) , P. major, Rumex crispus Linnaeus (1753) , Elytrigia repens Nevski (1933), Dactylis glomerata Linnaeus (1753) , Arrhenatherum elatius Presl & Presl (1819), Cichorium intybus Linnaeus (1753) , Carduus acanthoides Linnaeus (1753) , Trifolium repens Linnaeus (1753) , Ranunculus repens Linnaeus (1753) , Euphorbia palustris Linnaeus (1753) , Eryngium planum Linnaeus (1753) , Centaurea jacea Linnaeus (1753) , and Setaria pumila Roemer & Schultes (1817). [file peerj-14-21556-s005.jpg]

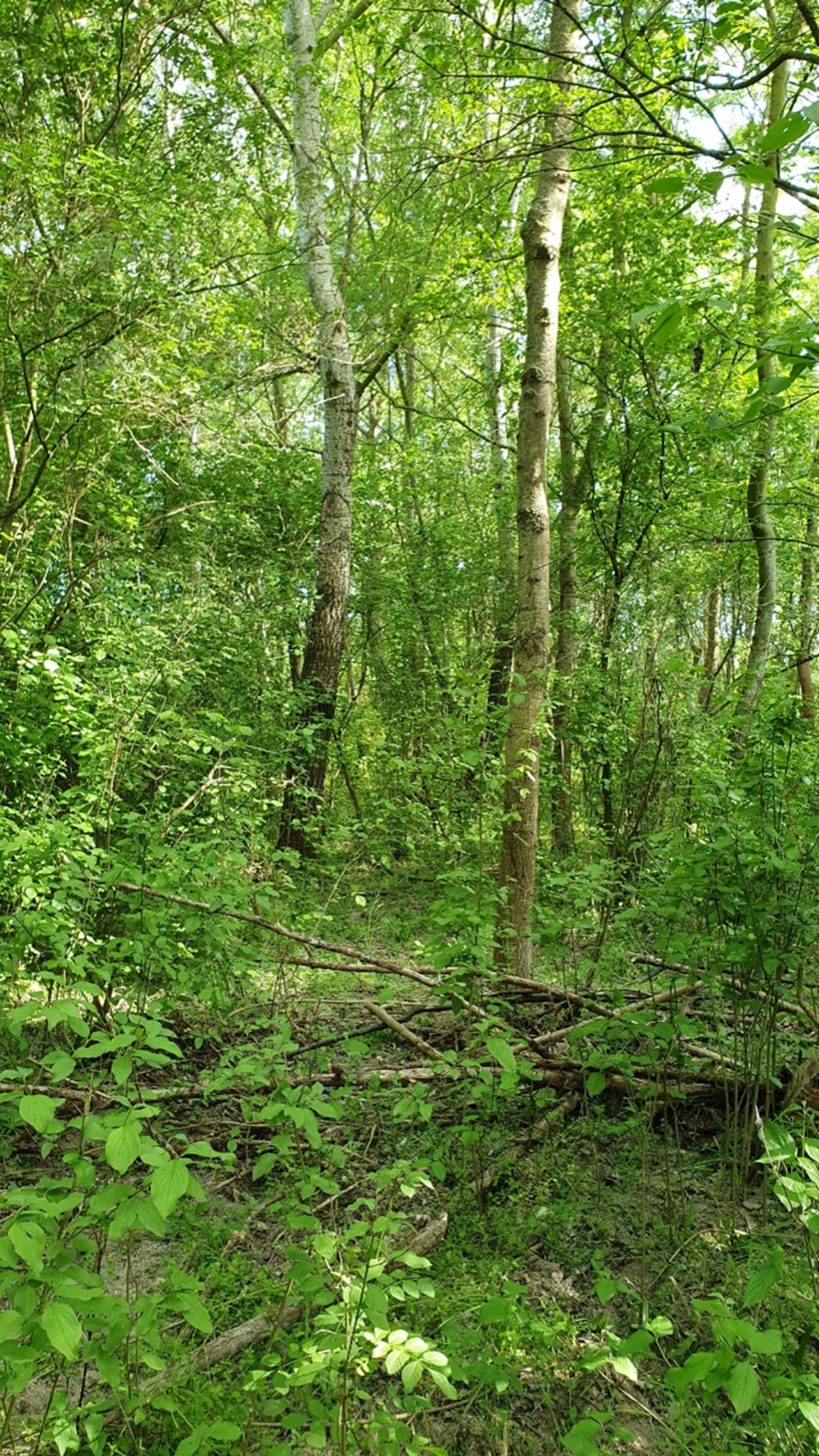

Supplement: Supplemental Information 6 — (SA6) = Pannonian poplar forest (forest habitat where revitalization measures were carried out = e xpansion of the branches of the Danube delta on the biotope, simulated flooding) ( Figure 16). Botanical description: The tree layer is formed by Populus nigra Linnaeus (1753) , P. alba, and S. alba. The herb layer was represented by the species U. dioica, R. caesius, G. aparine, R. canina, S. gigantea, D. glomerata, E. repens, and Stenactis annua Nees (1832). [file peerj-14-21556-s006.jpg]

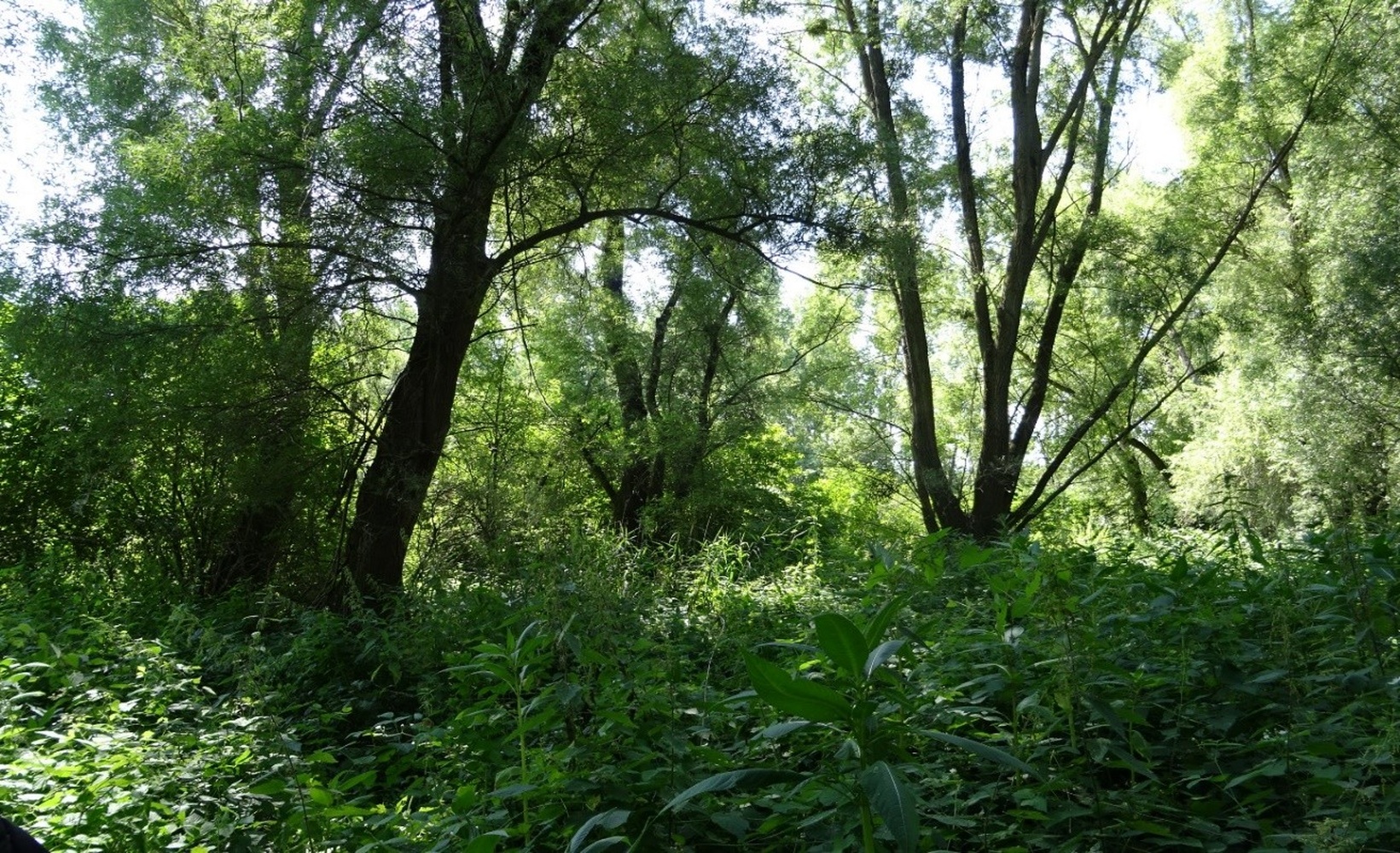

Supplement: Supplemental Information 7 — (SA7) = willow–poplar floodplain forest (forest habitat where revitalization measures were carried out = e xpansion of the branches of the Danube delta on the biotope, simulated flooding) ( Figure 17). Botanical description: The tree layer is composed of S. fragilis and S. alba. The herb layer was represented by the species U. dioica, I. glandulifera, S. gigantea, G. odoratum, P. officinalis, R. caesius, S. sylvatica, and L. maculatum. [file peerj-14-21556-s007.jpg]

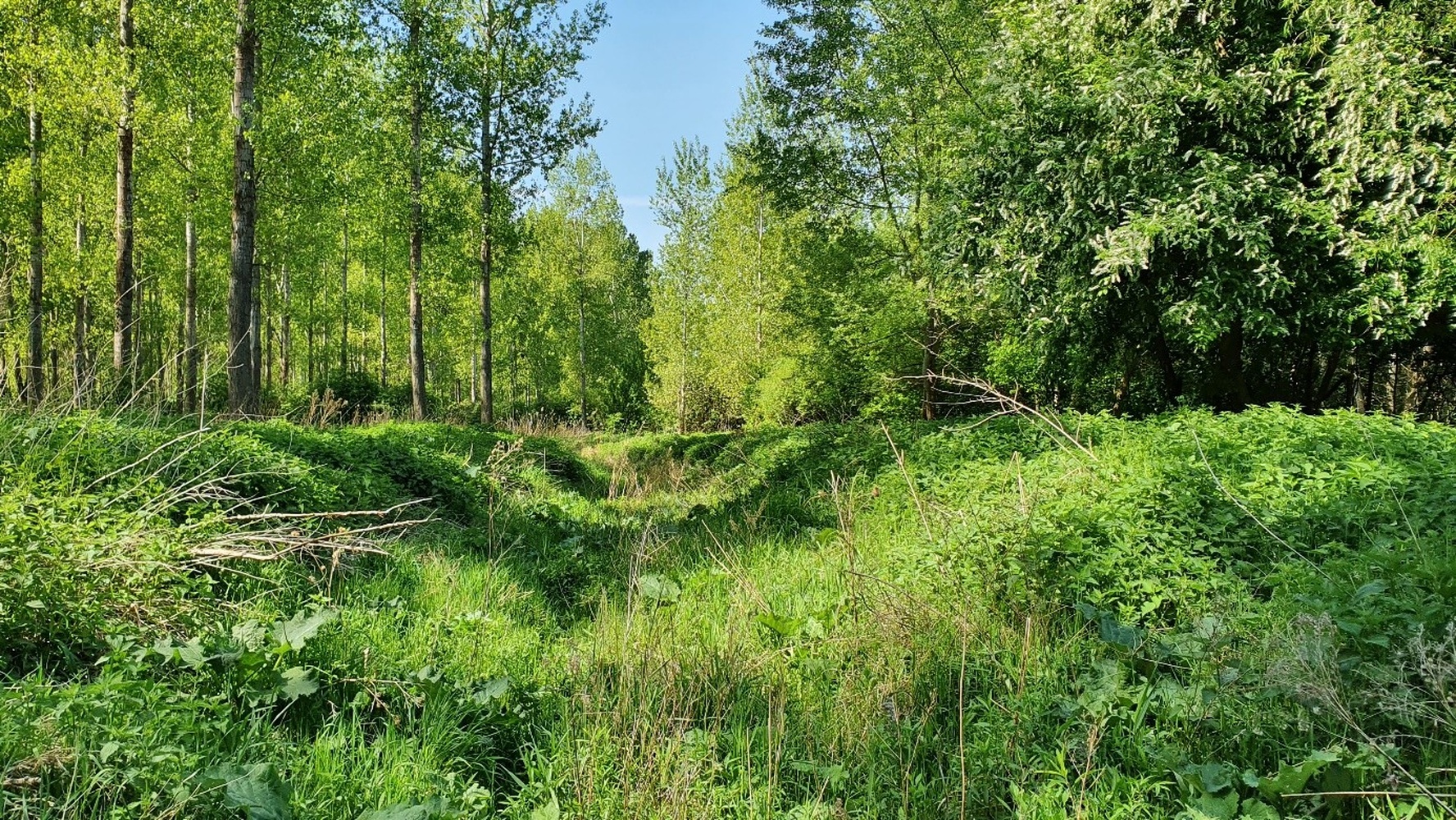

Supplement: Supplemental Information 8 — (SA8) = willow–poplar floodplain forest (forest habitat where revitalization measures were carried out = e xpansion of the branches of the Danube delta on the biotope, simulated flooding) ( Figure 18). Botanical description: The tree layer is composed of P. × canadensis, P. robusta, S. fragilis and Viscum album Linnaeus (1753). The shrub layer consists of S. nigra, Cornus sanguinea Linnaeus (1753) , and A. negundo. The herb layer was represented by the species Phragmites australis Steudel (1841), U. dioica, G. aparine, Ficaria bulbifera Holub (1961), Carduus crispus Linnaeus (1753) , Symphyotrichum lanceolatum Nesom (1995), R. caesius, I. glandulifera, Sparganium erectum agg. Linnaeus (1753), Arctium sp. Linnaeus (1753), H. lupulus, Galeopsis speciosa Miller (1768) and Cucubalus baccifer Linnaeus (1753). [file peerj-14-21556-s008.jpg]

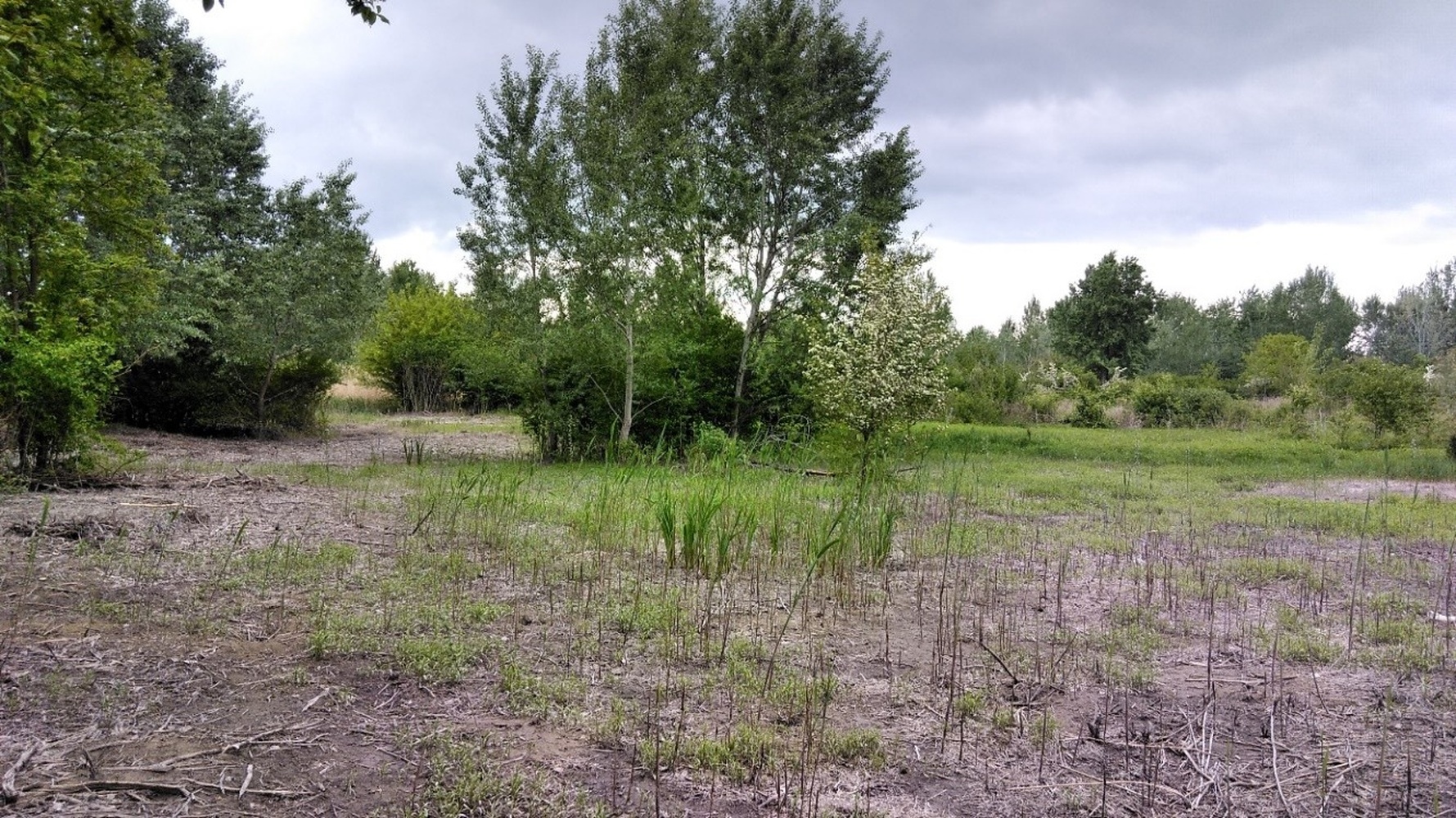

Supplement: Supplemental Information 9 — (SA9) = Reed communities of wetlands (the edge of a wetland directly connected to a forest habitat, where revitalization measures were carried out = e xpansion of the branches of the Danube delta on the biotope, simulated flooding) ( Figure 19). Botanical description: The tree layer is composed of S. alba, P. × canadensis, and A. negundo. The herb layer was represented by the species P. australis, U. dioica, Lythrum salicaria Linnaeus (1753), R. caesius, and S. gigantea. [file peerj-14-21556-s009.jpg]

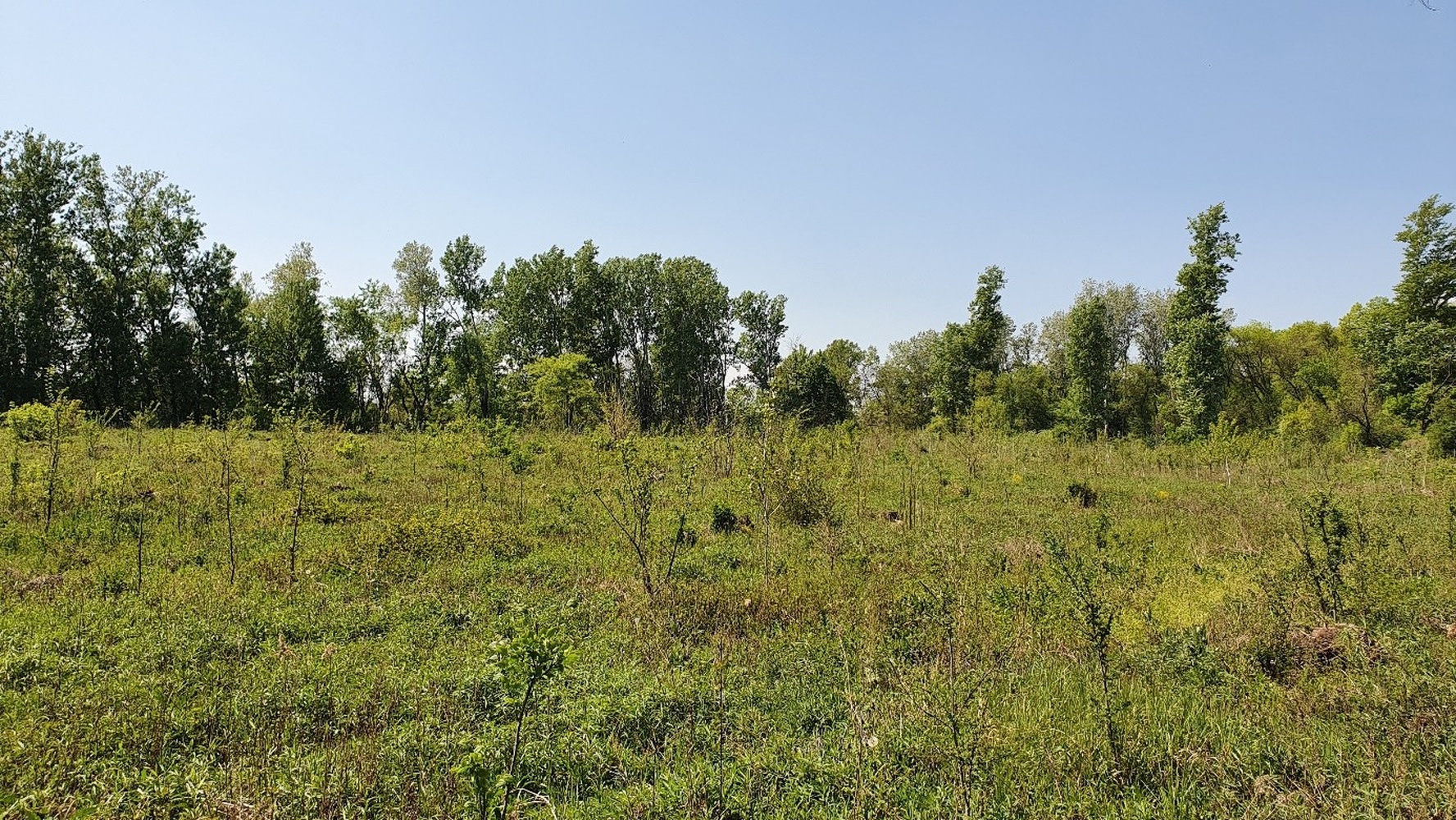

Supplement: Supplemental Information 10 — (SA10) = poplar nursery (planted poplar nursery where revitalization measures were carried out = e xpansion of the branches of the Danube delta on the biotope, simulated flooding) ( Figure 20). Botanical description: The study area was planted with P. alba and P. × canescens. It is a 2-year-old stand without a tree or shrub layer. The herb layer was represented by the species R. caesius, S. gigantea, P. alba, S. sanguinea agg., A. lanceolatus, U. dioica, Chenopodium album Linnaeus (1753), Symphytum officinale Linnaeus (1753), Stellaria media Villars (1789), Geum urbanum Linnaeus (1753), Erigeron annuus Persoon (1807) and E. repens. [file peerj-14-21556-s010.jpg]

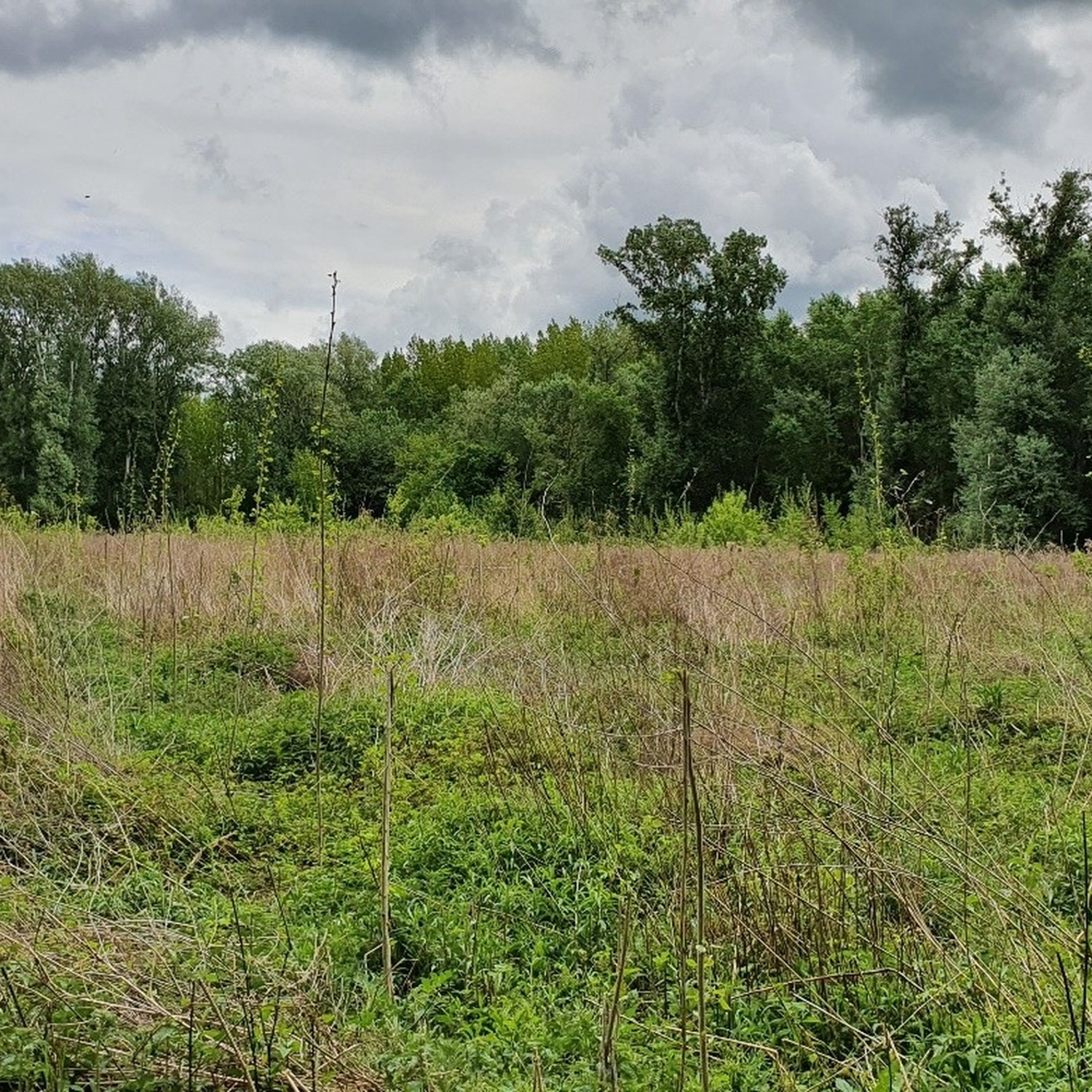

Supplement: Supplemental Information 11 — (SA11) = poplar nursery (planted poplar nursery where revitalization measures were carried out = e xpansion of the branches of the Danube delta on the biotope, simulated flooding) ( Figure 21). Botanical description: The study area was planted with P. alba and P. × canescens. It is a 2-year-old stand without a tree or shrub layer. The herb layer was represented by the species R. caesius, S. gigantea, P. alba, S. sanguinea agg., A. lanceolatus, U. dioica, Ch. album, S. officinale, S. media, G. urbanum, E. annuus and E. repens. [file peerj-14-21556-s011.jpg]

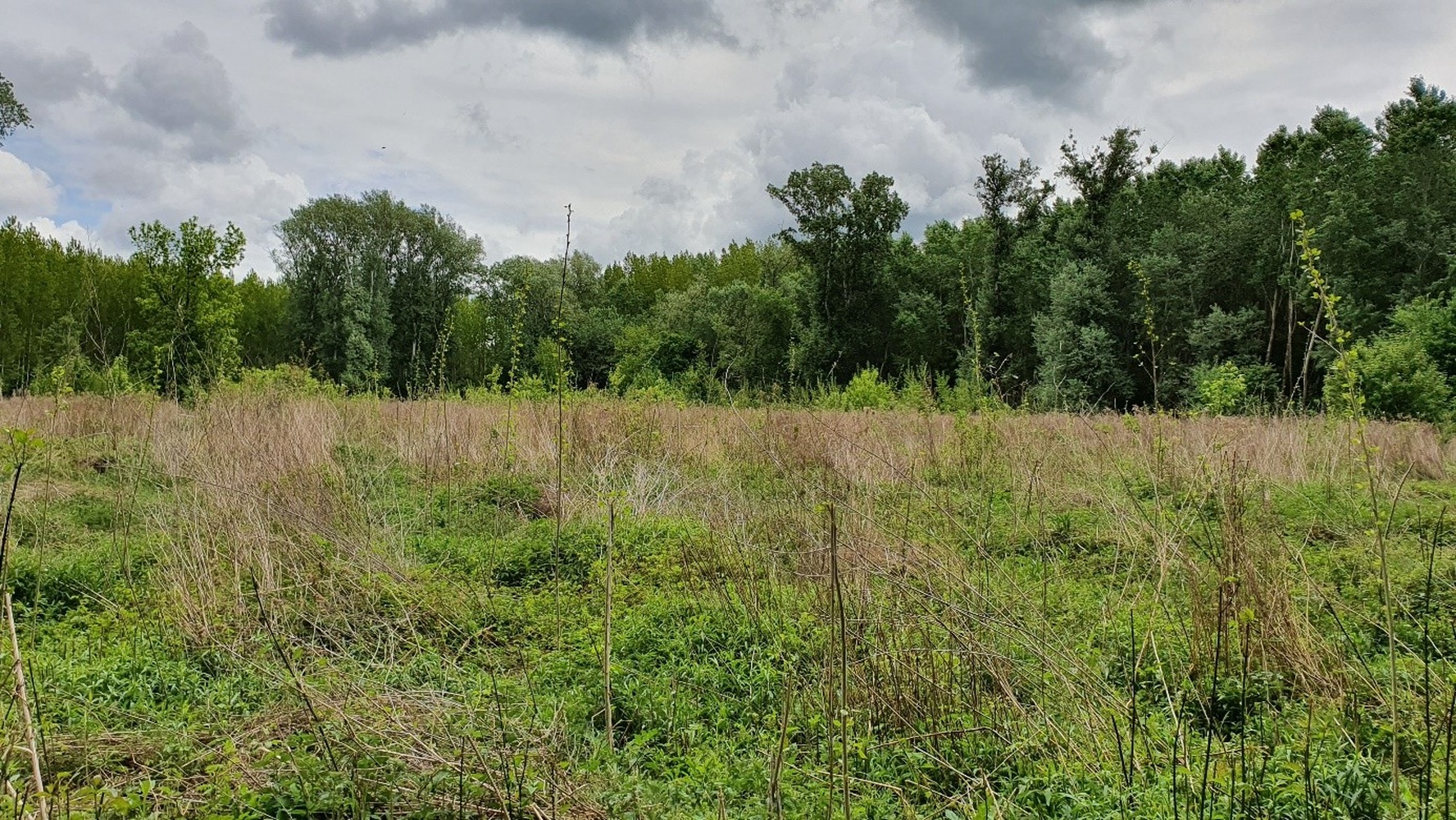

Supplement: Supplemental Information 12 — (SA12) = poplar nursery (planted poplar nursery where revitalization measures were carried out = e xpansion of the branches of the Danube delta on the biotope, simulated flooding) ( Figure 22). Botanical description: The study area was planted with P. alba and P. × canescens. It is a 2-year-old stand without a tree or shrub layer. The herb layer was represented by the species R. caesius, S. gigantea, P. alba, S. sanguinea agg., A. lanceolatus, U. dioica, Ch. album, S. officinale, S. media, G. urbanum, E. annuus and E. repens. [file peerj-14-21556-s012.jpg]

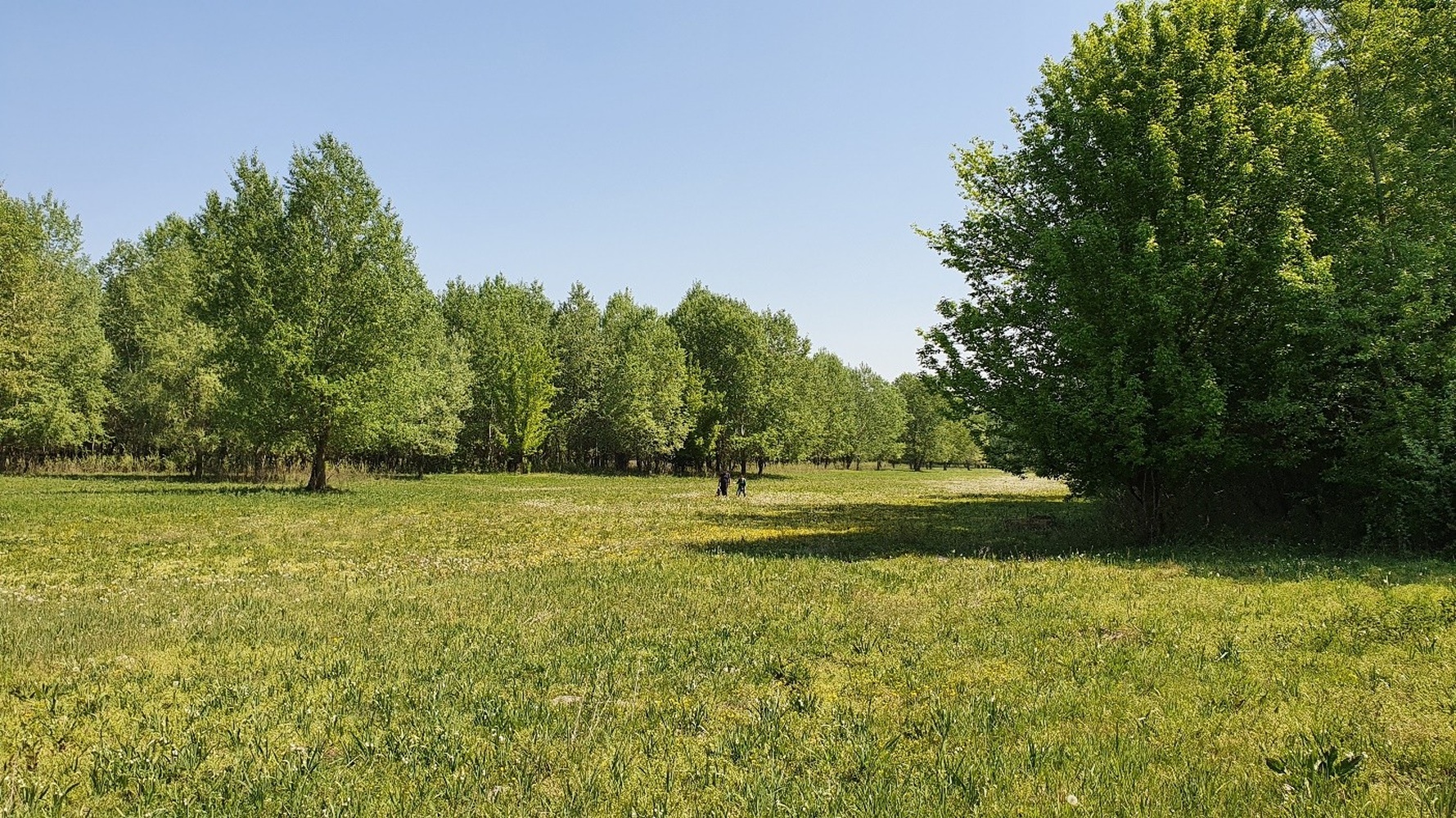

Supplement: Supplemental Information 13 — (SA13) = alluvial meadow (meadow habitat where revitalization measures were carried out = e xpansion of the branches of the Danube delta on the biotope, simulated flooding) ( Figure 23). Botanical description: Solitary individuals of Taraxacum sec. Ruderalia occurred within the study area. The herb layer was represented by the species A. millefolium, C. biennis, P. lanceolatum, P. major, E. repens, D. glomerata, A. elatius, C. intybus, T. repens, C. jacea, S. pumila, R. caesius, S. officinale, R. repens, R. acris, Leontodon autumnalis Oeder (1816), Thlaspi perfoliatum Linnaeus (1753), R. crispus, E. palustris, Iris pseudacorus Linnaeus (1753), and Galium boreale Linnaeus (1753). [file peerj-14-21556-s013.jpg]

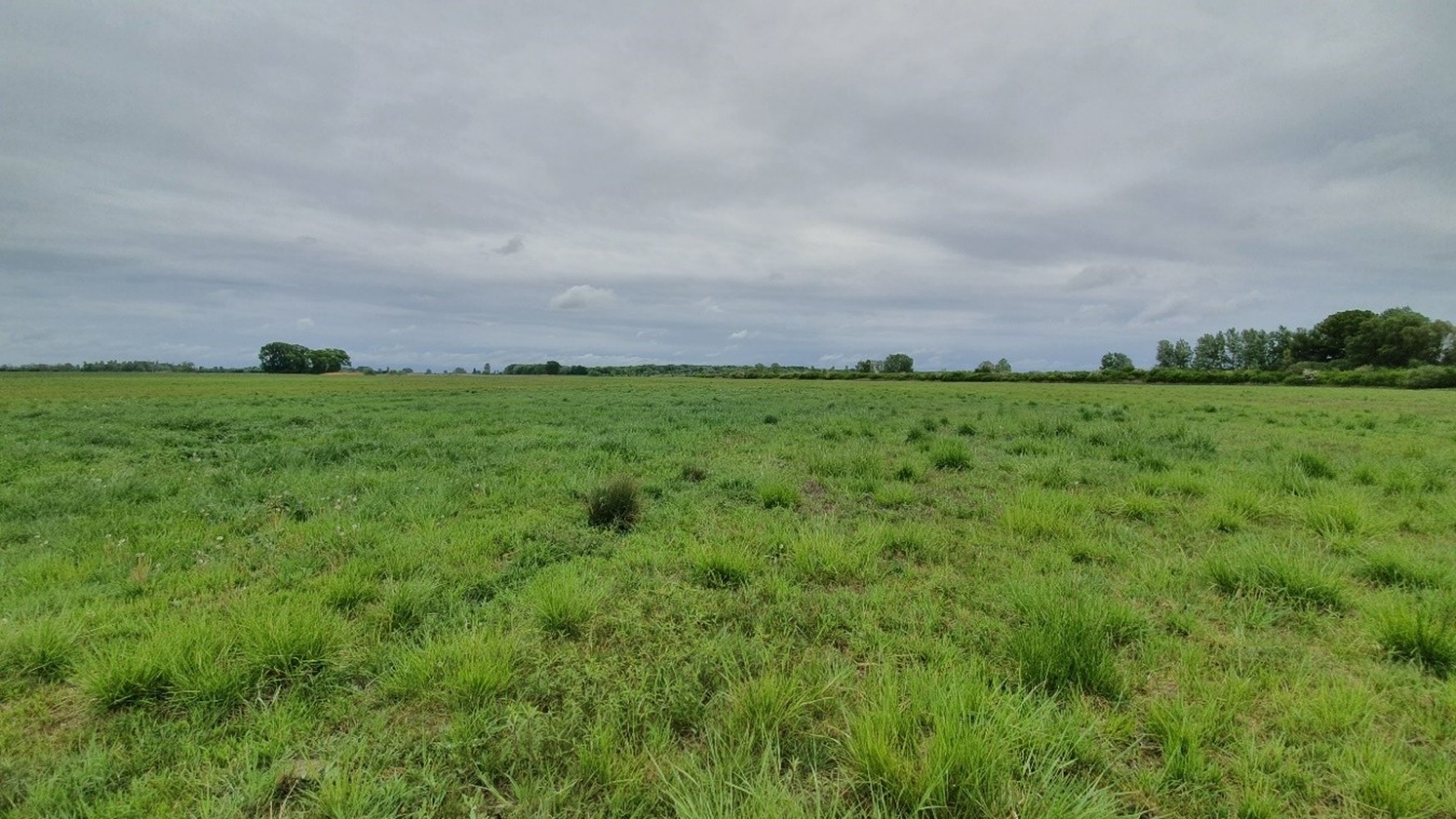

Supplement: Supplemental Information 14 — (SA14) = Lowland hay meadow (meadow habitat where revitalization measures were carried out = e xpansion of the branches of the Danube delta on the biotope, simulated flooding) ( Figure 24). Botanical description: The herb layer on the study area was represented by the species E. annuus, D. glomerata, C. intybus, Plantago media Linnaeus (1753), C. arvense, A. millefolium, E. repens, S. gigantea, Inula britannica Bieberstein (1808), and S. officinale. [file peerj-14-21556-s014.jpg]

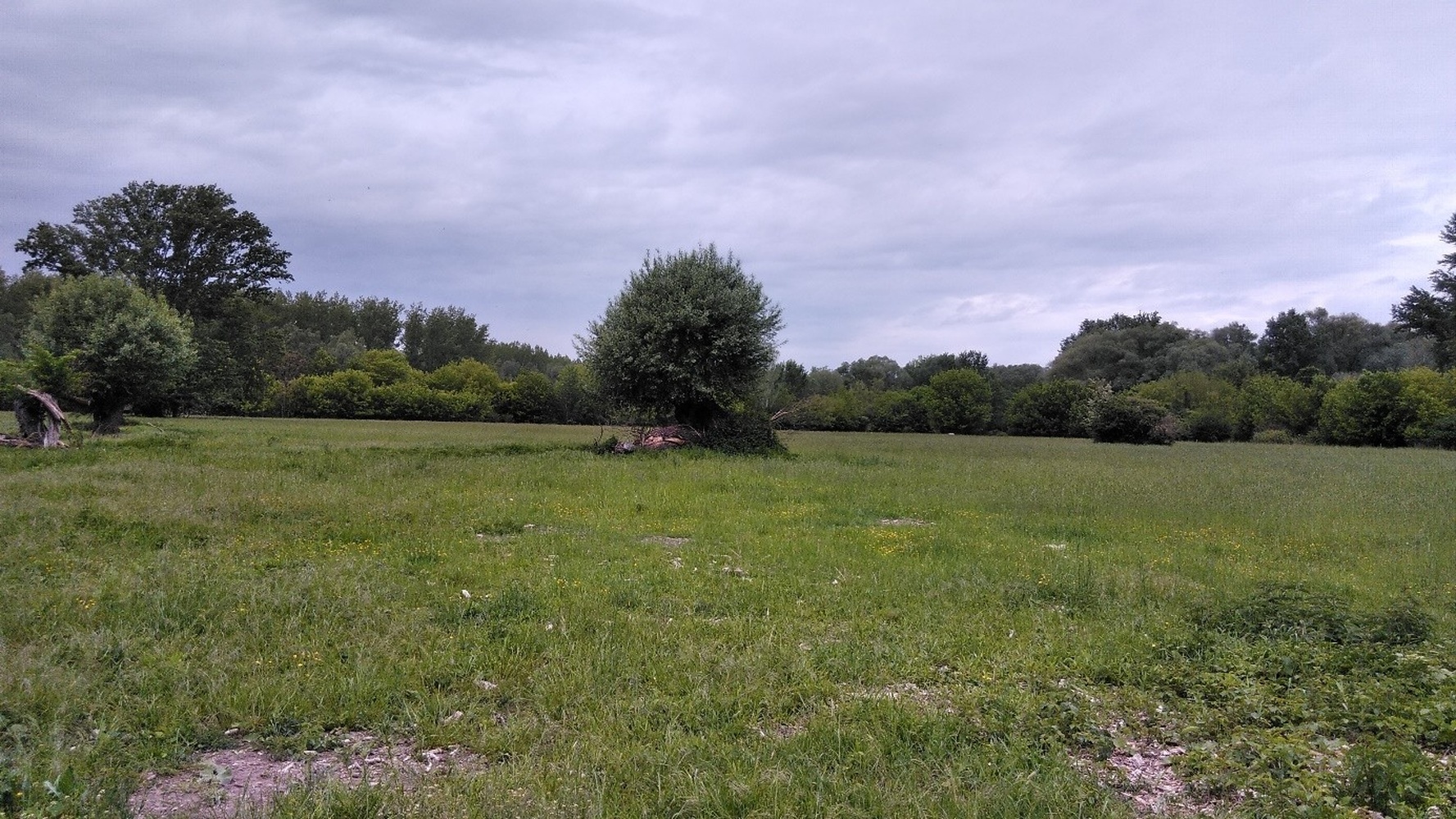

Supplement: Supplemental Information 15 — (SA15) = pasture (grassland where revitalization measures were carried out = expansion of the branches of the Danube delta on the biotope, simulated flooding, grazing by cattle) ( Figure 25). Botanical description: Solitary individuals of S. alba occurred within the study area. The herb layer was represented by the species C. arvense, C. acanthoides, A. lanceolatus, Artemisia campestris Linnaeus (1753), U. dioica, R. caesius, Agropyron repens Beauvois (1812), Lactuca serriola Linnaeus (1756), Tanacetum vulgare Linnaeus (1753), Glechoma hederacea Linnaeus (1753), Stenactis annua Nees (1832), A. millefolium, Poa pratensis Linnaeus (1753), P. media, Potentilla reptans Linnaeus (1753), Picris hieracioides Linnaeus (1753), G. parviflora, and Verbena officinalis Linnaeus (1753). [file peerj-14-21556-s015.jpg]
